# Comparison of the *in vivo* biodistributions of αvβ6-binding agents for PET imaging applications

**DOI:** 10.1016/j.omton.2026.201236

**Published:** 2026-05-14

**Authors:** Emma A. Swift, Stephen J. Paisey, Toby J. Phesse, John F. Marshall, Alan L. Parker, Rebecca J. Bayliss

**Affiliations:** 1 Division of Cancer and Genetics, School of Medicine, Cardiff University, Cardiff CF14 4XN, UK; 2 Wales Research and Diagnostic Positron Emission Tomography Imaging Centre, School of Medicine, Cardiff University, Cardiff CF14 4XN, UK; 3 European Cancer Stem Cell Research Institute, School of Biosciences, Cardiff University, Cardiff CF24 4HQ, UK; 4 The Peter Doherty Institute for Infection and Immunity, Melbourne, VIC 3000, Australia; 5 Centre for Tumor Biology, Barts Cancer Institute, Queen Mary University of London, London EC1M 6BQ, UK; 6 Wales Applied Virology Unit (WAVU), Cardiff University School of Medicine, Heath Park, Cardiff CF14 4XN, UK

**Keywords:** αvβ6 integrin, PET imaging, molecularly targeted radiotracers

## Abstract

Expression of the αvβ6 integrin is upregulated on multiple solid epithelial tumors while this integrin is largely absent on the healthy human epithelium. In the present study, we sought to compare the *in vivo* biodistributions of three αvβ6-selective agents that could be used for the positron emission tomography (PET)-based detection of malignancies expressing this integrin. A streptavidin-coupled αvβ6-binding peptide (streptavidin-A20), an anti-αvβ6 monoclonal antibody (620W.7) and an αvβ6-targeted protein, derived from a modified adenovirus 5 receptor-binding domain (Ad5.KO1.A20), were radiolabeled with [^89^Zr] and intravenously injected into mice bearing bilateral αvβ6-positive and -negative melanoma xenografts. Micro-PET imaging was performed at 0.33, 24, 48, 72, and (620W.7 group only) 144 h post-injection. Streptavidin-A20-based radiotracers preferentially accumulated in αvβ6-positive tumors, achieving >3.4:1 A375-β6:A375 activity ratios between 24 and 72 h post-injection, with off-target uptake restricted to the kidneys. 620W.7 exhibited roughly 2-fold greater accumulation in A375-β6 than A375 tumors, with lower levels of renal retention and greater activity detected in the liver and spleen. Despite having demonstrated selective binding to αvβ6-expressing cells *in vitro*, the Ad5.KO1.A20 protein failed to selectively traffic to tumors expressing this integrin *in vivo*. Nevertheless, streptavidin-A20 and 620W.7 demonstrated promise as αvβ6-selective agents for *in vivo* applications.

## Graphical abstract

**Figure:**
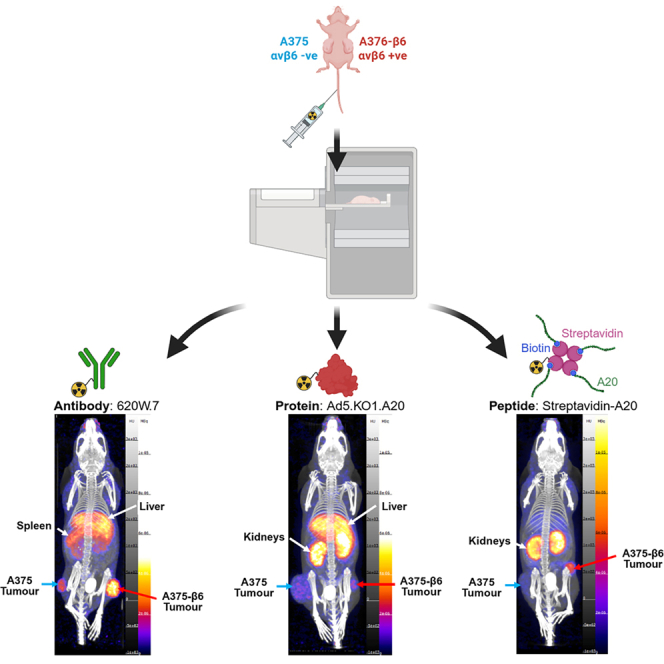

## Teaser

The *in vivo* biodistributions of ^89^Zr-labeled αvβ6-targeted agents were investigated in mice bearing bilateral αvβ6-positive and -negative human tumor xenografts by longitudinal micro-PET imaging. Peptide and antibody-based radiotracers were found to be effective at distinguishing αvβ6-expressing from non-expressing tumors *in vivo*, whereas an αvβ6-specific virus receptor-binding domain was not.

## Introduction

Integrins are a family of non-covalently associated, heterodimeric adhesion proteins that mediate crosstalk between cells and extracellular matrix (ECM) components. αvβ6 is an epithelial-restricted member of the integrin family,^1^ whose native ligands encompass several ECM glycoproteins,^2^ as well as latency-associated peptides linked to transforming growth factor (TGF)-β1 and TGF-β3 molecules in large latent complexes.^3^^,^^4^

Studies probing adult tissues for β6 expression have noted its conspicuous absence from the surface of most healthy epithelia. In contrast, β6 staining is detectable within the kidneys, lungs, and skin of fetal tissues, suggesting a physiological role for αvβ6 in organogenesis, with subsequent downregulation of integrin expression following tissue differentiation.^1^ However, *de novo* expression of αvβ6 has been reported to occur in epithelial carcinomas arising in several different anatomical locations, including those affecting the oral squamous epithelium,^5^ pancreas,^6^ colon,^7^ stomach,^8^ ovaries,^9^ and bile duct.^10^ Integrin upregulation in these settings is generally associated with poorer outcomes.^7^^,^^8^^,^^9^^,^^10^

Several therapies seeking to exploit the cancer-associated expression profile of αvβ6 have emerged in recent years, including αvβ6-targeted CAR T cells,^11^^,^^12^ oncolytic virotherapy platforms,^13^^,^^14^ and immunoliposomes loaded with cytotoxic cargoes.^15^ The greatest responses to these therapeutics can be expected to occur in patients whose tumors express high levels of αvβ6. Molecularly targeted PET imaging offers a non-invasive means through which individuals may be stratified for treatment with such αvβ6-selective therapeutics.

PET-based detection of αvβ6-expressing malignancies has previously been facilitated through the use of radiolabeled integrin-binding peptides. This includes those based on the linear A20 peptide sequence (NAVPNLRGDLQVLAQKVART) derived from the αvβ6-engaging VP1 protein of the foot-and-mouth disease virus,^16^^,^^17^ as well as peptides selected from sunflower trypsin inhibitor-1 scaffold-guided phage display libraries,^18^^,^^19^ knottins,^20^ and head-to-tail cyclized peptides.^21^ These agents have shown promise in their ability to detect αvβ6-expressing pathologies in preclinical studies and small first in-human clinical trials.^16^^,^^17^^,^^18^^,^^19^^,^^20^^,^^21^^,^^22^^,^^23^^,^^24^ Nevertheless, peptide tracers suffer from a propensity to be reabsorbed within the kidneys, resulting in dose-limiting nephrotoxicities.^25^ Furthermore, several of the non-backbone cyclized αvβ6-binding peptide variants examined to date have been shown to accumulate in the GI tract,^18^^,^^20^^,^^22^^,^^23^^,^^24^ limiting their capacity to accurately delineate tumors arising within this region.^26^

In the present study, we sought to compare the *in vivo* biodistribution of a streptavidin-coupled A20 peptide-based radiotracer (streptavidin-A20) with the uptake profiles of two other classes of αvβ6-targeted biologics. These consisted of a monoclonal anti-αvβ6 antibody (620W.7) and a protein derived from the primary receptor-binding domain of an αvβ6-selective adenovirus 5 virotherapy platform.^14^ All three agents were labeled with the long half-life zirconium-89 [^89^Zr] radionuclide and injected intravenously into mice bearing bilateral αvβ6-positive and -negative melanoma tumor xenografts. This facilitated evaluation of their uptake kinetics and biodistribution profiles in a longitudinal microPET imaging study.

## Results

### Overview of αvβ6-targeted biologics

**Figure 1:**
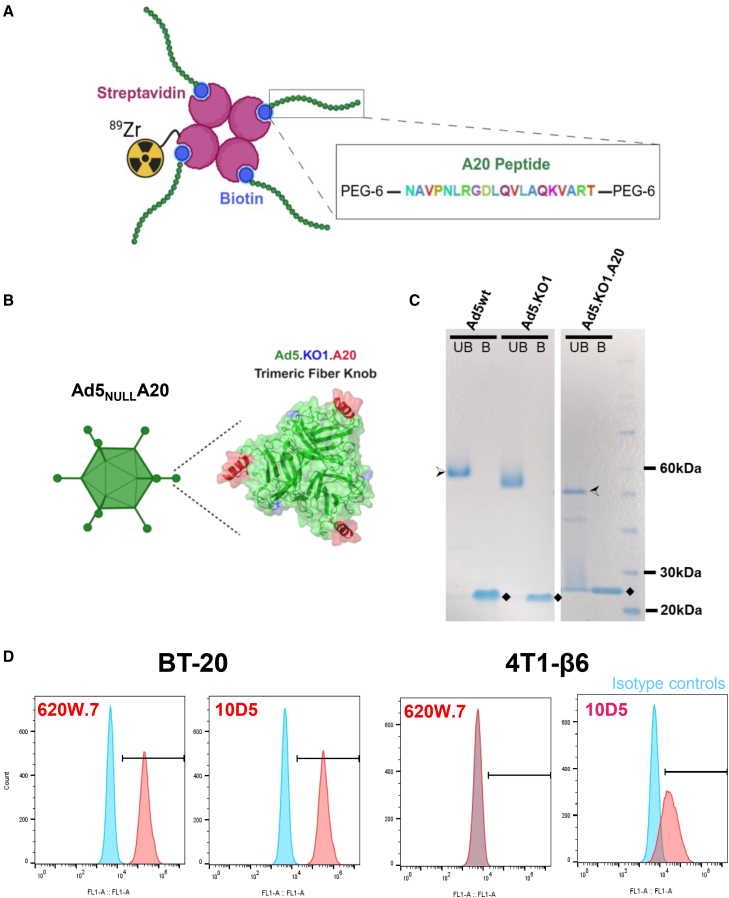
Overview of αvβ6-targeted biologics (A) Schematic overview of the indirect radiolabeling approach adopted for the A20 peptide-based tracer. (B) Cartoon depicting the location of the fiber knob domain within Ad5_NULL_-A20 virions (left), alongside a model for the trimeric Ad5.KO1.A20 protein. The A20 peptide insert is depicted in red, and S408E, P409A substitution mutations that ablate CAR binding (collectively referred to as KO1) are shown in blue. (C) SDS-PAGE assessment of recombinant fiber knob trimerization. Half-filled arrowheads denote bands corresponding to trimeric forms of the relevant fiber knob, while monomers are indicated by black diamonds. UB, un-boiled; B, boiled; wt, wildtype; PEG, polyethylene glycol. The full gel image is provided in Figure S1B. (D) Representative flow cytometry histograms depicting staining of BT-20 cells (which express human αvβ6) and 4T1-β6 cells (expressing the equivalent murine isoform) with the 620W.7 antibody. A second commercially available isoform cross-reactive anti-αvβ6 antibody (clone 10D5) was used as a control.

Previous investigations into the αvβ6-binding A20 peptide have revealed that its retention within tumors and *in vivo* pharmacokinetic profile can be improved by the addition of polyethylene glycol (PEG) moieties at both its N and C terminus.^27^ The A20 peptide utilized in the present study was therefore synthesized with biterminal PEG-6 linkers. Furthermore, we incorporated an N-terminal biotin modification into our peptide-based radiotracer design to facilitate A20 coupling to succinylated ^89^Zr-streptavidin in an indirect radiolabeling approach (Figure 1A). This was adopted to minimize levels of renal retention by increasing the overall molecular weight of the radiotracer. When mixed in a 4:1 peptide: streptavidin molar ratio, the predicted size of the resulting complex was 64.9 kDa.

Secondly, we generated an αvβ6-targeted protein derived from the receptor-binding domain of a modified adenovirus 5 virotherapy platform. The oncolytic Ad5_NULL_-A20 virus has been engineered to selectively infect and lyse αvβ6-expressing tumors.^14^ Its αvβ6-dependent mechanism of cell entry is dependent upon the A20 peptide, which is inserted into a permissive loop of the virus fiber knob domain (Figure 1B). When combined with two point mutations (S408E, P409A, collectively referred to as KO1) that ablate the high affinity native interaction of this domain with the coxsackie and adenovirus receptor (CAR) (Figure S1A),^28^ this results in αvβ6 integrin-dependent virus entry.^14^^,^^29^ We hypothesized that the knob domain of this virus could serve as a useful αvβ6-binding agent when expressed independently of the whole viral vector. As such, we generated recombinant versions of this protein (referred to as Ad5.KO1.A20), alongside an untargeted control domain lacking the A20 insert (Ad5.KO1). Both proteins retained the native capacity of adenoviral fiber knob proteins to trimerise (Figure 1C), providing three available αvβ6-binding sites per Ad5.KO1.A20 trimer.

The final class of αvβ6-binding biologic examined in the present study consisted of an anti-αvβ6 monoclonal antibody (clone 620W.7). 620W.7 recognizes the human αvβ6 integrin heterodimer but lacks cross-reactivity with the equivalent murine isoform (Figure 1D).

### *In vitro* evaluation of targeted agent binding to αvβ6

**Figure 2:**
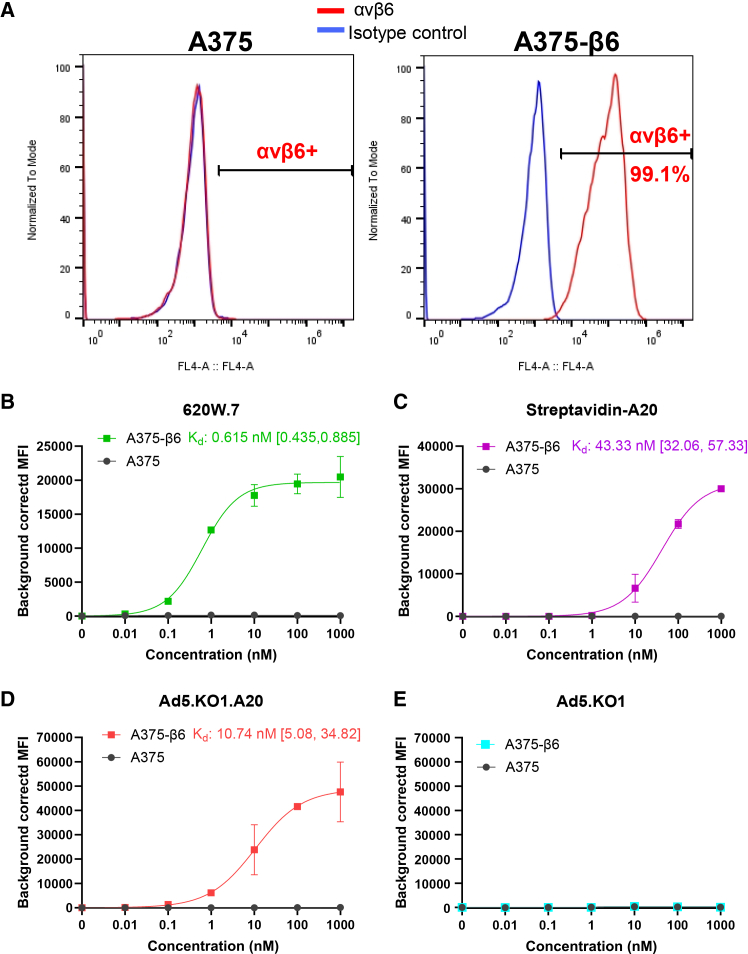
*In vitro* evaluation of αvβ6-targeted agent binding to A375 and A375-β6 cells (A) Representative flow cytometry histograms depicting staining of A375 and A375-β6 cells for αvβ6. Increasing concentrations of the (B) 620W.7 antibody, (C) streptavidin-A20 complex (prepared in a 1:1 streptavidin:A20 molar ratio), (D) Ad5.KO1.A20 knob protein, and (E) Ad5.KO1 recombinant fiber knob were incubated with A375 and A375-β6 cells. Median fluorescence intensity values (MFI) were determined in triplicate for each concentration, and data background corrected to the MFI of the 0 nM condition. Non-linear regression curves were fitted using the specific binding with Hill slope equation in GraphPad Prism (v.10.4.1) and Kd values are reported alongside their 95% confidence intervals (profile likelihood). Data are presented as mean ± standard deviation.

Serial dilutions of each of the candidate αvβ6-targeted agents were incubated with A375 cells, which lack αvβ6 but reportedly express other related members of the integrin heterodimer family,^30^^,^^31^ as well as a matched A375-β6 line engineered to overexpress the human αvβ6 isoform (Figure 2A).

All three targeted agents demonstrated clear binding to the A375-β6 cell line at nanomolar concentrations without detectable binding to A375 cells, consistent with their selective, high affinity recognition of αvβ6 (Figures 2B–2D). As expected, the untargeted Ad5.KO1 protein didn’t bind to either cell line (Figure 2E). Furthermore, the αvβ6-binding capacity of these agents was not unduly disrupted by either exposure to murine serum (Figure S2) or conjugation to the deferoxamine chelator required for their subsequent ^89^Zr-radiolabelling (Figure S3).

### *In vivo* biodistribution of αvβ6-targeted agents

The three αvβ6-targeted agents and untargeted Ad5.KO1 control protein were labeled with [^89^Zr] to high radiochemical yields and purities as assessed by radio-TLC (Figure S4) and radio-SDS-PAGE (Figure S5). 2 MBq of each tracer was subsequently injected into the tail veins of CD-1 nude mice bearing A375 and A375-β6 tumors over their left and right hip regions, respectively.

### ^89^Zr-streptavidin-A20

**Figure 3:**
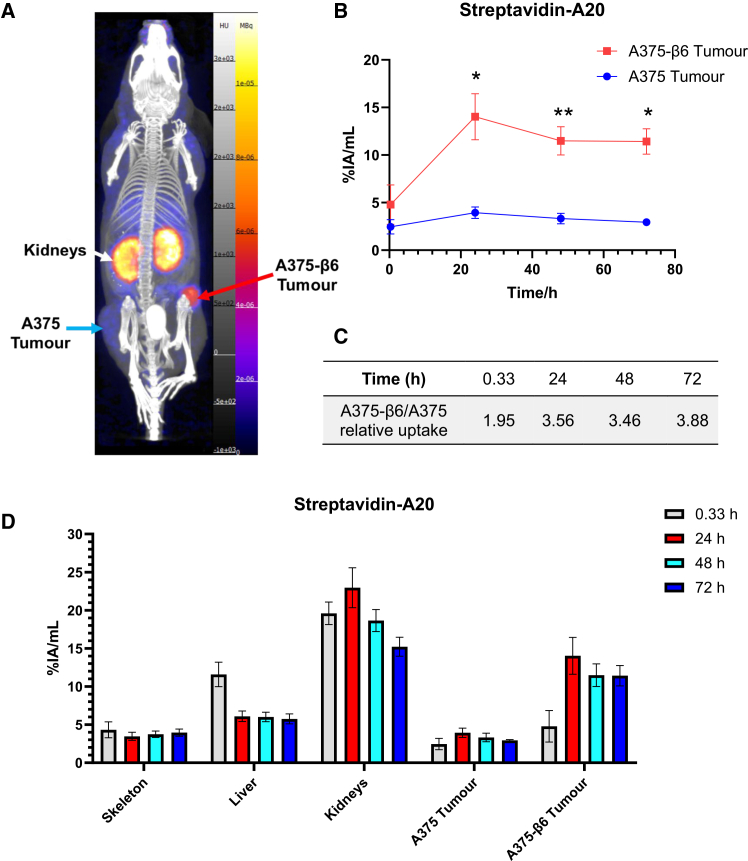
*In vivo* biodistribution of ^89^Zr-streptavidin-A20 radiotracers (A) Representative overlaid maximum intensity projection (MIP) views of PET/CT scans for a mouse injected with ^89^Zr-streptavidin-A20, obtained after 24 h. (B) Comparison of radioactivity levels in A375-β6 and A375 tumors for ^89^Zr-streptavidin-A20-injected mice (0.33–48 h: *n* = 4 for both groups, 72 h: *n* = 3 for A375-β6 and *n* = 2 for A375 tumor groups). (C) Relative uptake in A375-β6 tumors over A375 tumors across the four imaging time points. (D) Radiotracer uptake in organs of interest for ^89^Zr-streptavidin-A20 injected mice (tumor n numbers as in (B), all other regions 0.33–48 h: *n* = 6, 72 h: *n* = 4). Data are presented as mean ± standard deviation. ∗adjusted *p* value<0.0332, ∗∗ adjusted *p* value<0.0021.

^89^Zr-streptavidin-A20 radiotracers demonstrated excellent selectivity in trafficking to αvβ6-positive tumors while minimally accumulating in A375 xenografts. Across the 24–72 h imaging window, radioactivity in A375-β6 tumors remained >3.4 times that in αvβ6-negative A375 tumors, peaking at 14.03 ± 2.42% IA/mL after 24 h (Figures 3A–3C).

The major site of off-target uptake for ^89^Zr-streptavidin-A20 was within the kidneys. Activity within this site peaked at 22.96 ± 2.62% IA/mL 24 h post-injection, before gradually declining to 15.22 ± 1.24% IA/mL by the final imaging time point. Comparatively little radioactivity was detected within the livers of these mice, remaining around 6% IA/mL, with no evidence of tracer accumulation in the GI tract or spleen (Figures 3A and 3D).

### ^89^Zr-620W.7

**Figure 4:**
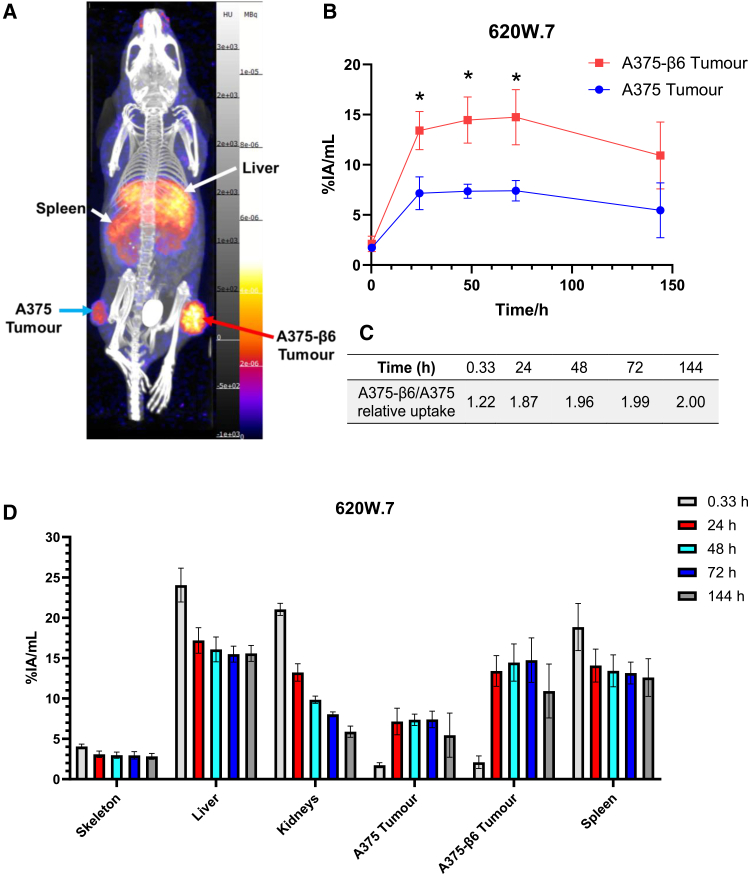
*In vivo* biodistribution of ^89^Zr-620W.7 (A) Representative overlaid MIP views of PET/CT scans for a mouse injected with ^89^Zr-620W.7, obtained after 72 h. (B) Comparison of radioactivity in A375-β6 and A375 tumors for ^89^Zr-620W.7-injected mice (0.33h: *n* = 3 for A375-β6 and *n* = 4 for A375 tumor groups, 24–144 h: *n* = 4 for both groups). (C) Relative uptake of ^89^Zr-620W.7 in A375-β6 tumors over A375 tumors. (D) Radiotracer uptake in organs of interest for ^89^Zr-620W.7-injected mice (tumor *n* numbers as in (B), all other regions *n* = 5). Data are presented as mean ± standard deviation. ∗adjusted *p* value<0.033.

Similar to ^89^Zr-Streptavidin-A20 tracers, ^89^Zr-620W.7 preferentially accumulated in A375-β6 tumors over A375 xenografts (Figures 4A–4C). Indeed, %IA/mL values for ^89^Zr-620W.7 in A375-β6 tumors were slightly higher than those measured for the A20 peptide-based radiotracer at the 48 h and 72 h time points (Figure S6), peaking 72 h post-injection at 14.75 ± 2.75% IA/mL. However, uptake in the A375 tumors was also increased for ^89^Zr-620W.7, resulting in only approximately 2:1 A375-β6:A375 tumor activity ratios (Figure 4C).

**Figure 5:**
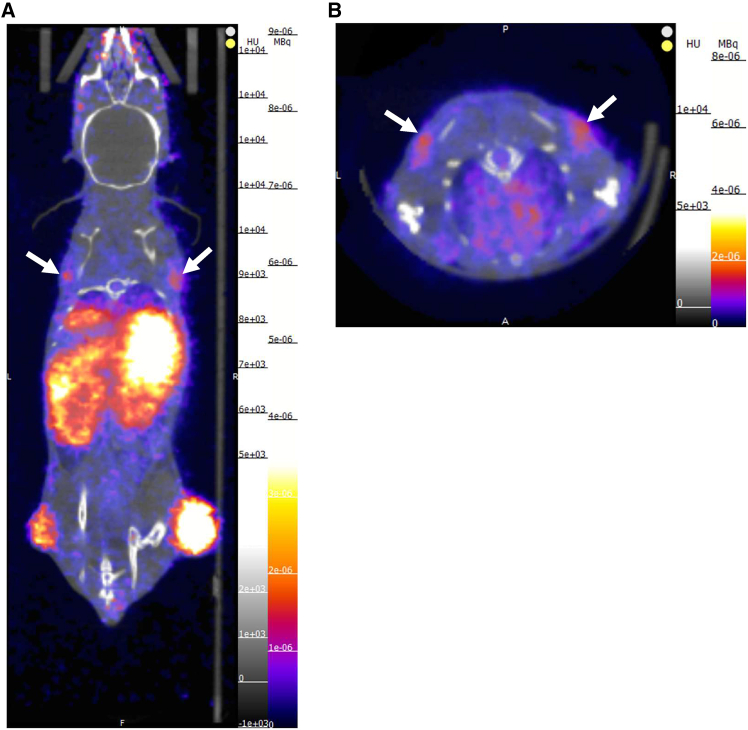
Axillary lymph node uptake of ^89^Zr-620W.7 (A) Coronal and (B) transverse sections of an ^89^Zr-620W.7-injected mouse, obtained after 48 h. White arrows denote radiotracer uptake within axillary lymph nodes.

Though initially high, radioactivity levels in the kidneys of ^89^Zr-620W.7-injected mice dropped rapidly following the first scan, suggestive of radiotracer washout from this site. In contrast, roughly 15%–17.2% IA/mL was retained within the livers of these mice from 24 to 144 h. Similar levels of activity were also detected in their spleens (Figure 4D), with further focal accumulation of ^89^Zr-620W.7 visible within the shoulder regions of mice, thought to correspond to uptake into axillary lymph nodes (Figures 5A and 5B).

### ^89^Zr-Ad5.KO1.A20

**Figure 6:**
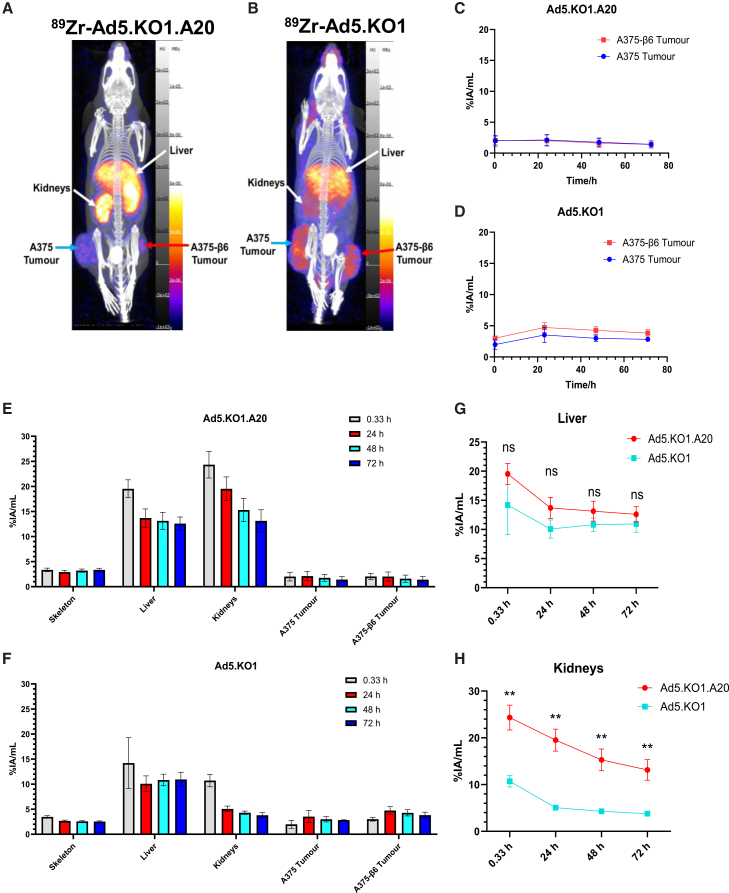
*In vivo* biodistribution of adenoviral fiber knob-based radiotracers Representative overlaid MIP views of PET/CT scans for mice injected with (A) ^89^Zr-Ad5.KO1.A20 and (B) ^89^Zr-Ad5.KO1, obtained after 24 h. Comparison of radioactivity in A375-β6 and A375 tumors for (C) ^89^Zr-Ad5.KO1.A20-injected mice (0.33 h: *n* = 3 for A375 and *n* = 2 for A375-β6 tumor groups, 24–72 h: *n* = 3 for both groups), and (D) ^89^Zr-Ad5.KO1-injected mice (0.33–24 h: *n* = 3 for A375 and *n* = 4 for A375-β6 tumor groups, 48–72 h: *n* = 2 for A375 and *n* = 3 for A375-β6). Radiotracer uptake in organs of interest for (E) ^89^Zr-Ad5.KO1.A20-injected mice (tumor *n* numbers as in C all other regions, *n* = 4) and (F) ^89^Zr-Ad5.KO1-injected mice (tumor *n* numbers as in D all other regions, 0.33–24 h: *n* = 5, 48–72 h: *n* = 4). Comparison of levels of (G) hepatic and (H) renal tracer uptake for the ^89^Zr-Ad5.KO1 (blue) and ^89^Zr-Ad5.KO1.A20 (red) radiotracers (*n* numbers as in C and D). Data are mean ± standard deviation. ∗∗ adjusted *p* value < 0.0021, ns: no significant difference

In contrast to the other two αvβ6-targeted agents, ^89^Zr-Ad5.KO1.A20 radiotracers did not traffic to αvβ6-expressing tumors. %IA/mL values were <2.5% for this tracer in both A375 and A375-β6 tumors throughout the study, similar to the untargeted Ad5.KO1 control protein (Figures 6A–6D).

Radioactivity was detected in both the liver (∼13% IA/mL for scans at 24–72 h) and kidneys (starting at 24.32 ± 2.65% IA/mL, decreasing to 13.13 ± 2.21% IA/mL by 72 h) of ^89^Zr-Ad5.KO1.A20-injected mice (Figure 5E). Interestingly, while levels of hepatic retention were similar between the ^89^Zr-Ad5.KO1 and ^89^Zr-Ad5.KO1.A20 groups (Figure 6G), the αvβ6-targeted protein exhibited significantly greater kidney uptake (Figure 6H).

*Ex vivo* dosimetry measurements corresponded well to uptake values calculated from PET imaging scans (Figure S7).

### Excretion rates

High levels of radioactivity were observed within the intestines and bladders of knob protein-injected mice during initial microPET scans, whereas this was not a feature of the other two αvβ6-targeted agents (Video S1). We therefore examined whether radiotracer excretion via these routes might underpin the failure of ^89^Zr-Ad5.KO1.A20 to traffic to αvβ6-expressing tumors *in vivo*.

**Figure 7:**
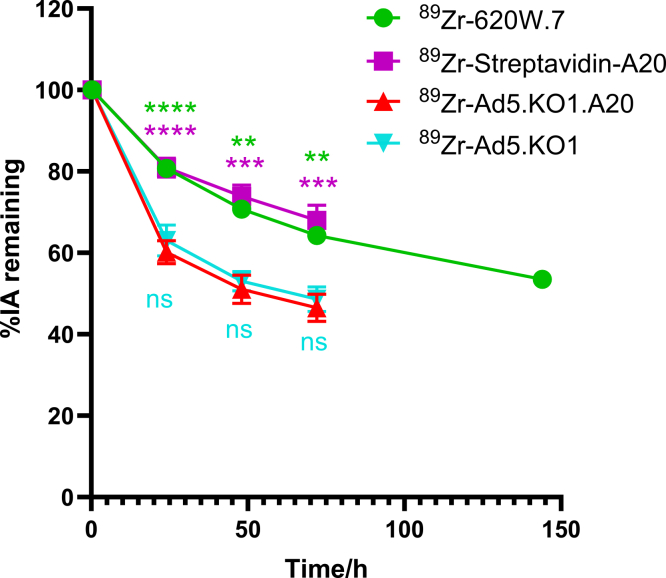
Excretion rates for αvβ6-targeted agents The average radioactivity remaining at each time point was calculated by summing the total radioactivity present within whole body PET scans and expressed as a decay-corrected percentage of the activity present in initial scans for mice injected with ^89^Zr-620W.7 (green, *n* = 5), ^89^Zr-streptavidin-A20 (purple, 0.33–48 h: *n* = 6, 72 h: *n* = 4), ^89^Zr-Ad5.KO1.A20 (red, *n* = 4) and ^89^Zr-Ad5.KO1 (blue, 0.33–24 h: *n* = 5, 48–72 h: *n* = 4). Data are presented as mean ± standard deviation. ∗∗adjusted *p* value<0.0021, ∗∗∗adjusted *p* value < 0.0002, ∗∗∗∗adjusted *p* value < 0.0001, ns: no significant difference (compared to ^89^Zr-Ad5.KO1.A20 group).

While total radioactivity within scans of Ad5.KO1- and Ad5.KO1.A20-injected mice decreased faster than for the other two αvβ6-targeted agents, 46.52 ± 3.35% of the initial dose remained by the final time point (Figure 7). Hence, excretion of the Ad5.KO1.A20-based radiotracer alone cannot wholly account for its lack of uptake into A375-β6 tumors.

## Discussion

The contribution of αvβ6 upregulation to the progression and metastasis of several aggressive epithelial carcinomas has resulted in significant interest in the potential to exploit its expression as a tumor-specific vulnerability in anti-cancer therapeutic approaches.^7^^,^^8^^,^^9^^,^^10^ The present study outlines the evaluation of three αvβ6-binding biologics that may be used as molecularly targeted agents for the non-invasive diagnosis and monitoring of tumors expressing this integrin.

Our findings solidify the promise of A20 peptide-based radiotracers to serve as the basis for effective αvβ6-targeted PET imaging tracers. Consistent with previous studies,^16^^,^^22^^,^^23^ the A20 peptide-based radiotracer demonstrated an extremely promising *in vivo* biodistribution, rapidly accumulating in αvβ6-expressing tumors and exhibiting >3.4-fold greater accumulation in these compared to αvβ6-negative tumors over a 3-day imaging period (Figure 3).

The kidneys were the primary site of streptavidin-A20 off-target uptake (Figure 3D). Significantly greater renal retention was also observed when comparing the biodistribution of the Ad5.KO1.A20 fiber knob to the untargeted Ad5.KO1 control, which was not observed in the liver (Figures 6G and 6H). Endogenous expression of αvβ6 in murine kidneys has been shown to be negligible, while A20 peptide trapping within this site has previously been prevented by co-injection of a scrambled version of this sequence.^16^^,^^32^ This suggests that renal uptake of A20-containing tracers is unlikely to occur via target-specific mechanisms and instead relates to its physicochemical properties. Notably, the levels of ^89^Zr-streptavidin-A20 renal trapping observed here do compare favorably to those reported previously for A20-based radiotracers, particularly those making use of similar bi-terminally PEGylated peptide variants.^16^^,^^27^ This may be reflective of the increased molecular weight of the tracer used here, achieved via peptide complexation to radiolabeled streptavidin, which was further succinylated to reduce its isoelectric point and propensity to be reabsorbed by proximal tubular cells.^33^ Additional studies are required to determine the half-life and other specific pharmacokinetic parameters for the streptavidin-A20 tracer developed here and compare these values to existing αvβ6-binding peptides. Should our indirect labeling method prove to provide favorable pharmacokinetic properties, this could be a broadly applicable strategy for reducing the nephrotoxicity risk associated with the use of low molecular weight peptides as radiopharmaceuticals.^25^ Indeed, this strategy, based entirely on commercially available agents, has the potential to work for any biotinylated peptide and could provide an alternative to antibody labeling strategies when antibodies are not available or targets in the liver or spleen are of interest.

The anti-αvβ6 monoclonal antibody 620W.7 was also effective in delineating A375-β6 tumors in our preclinical imaging study, with maximal accumulation after 72 h (Figure 4). However, while ∼2-fold lower than the activity level in matched A375-β6 tumors, weak uptake of ^89^Zr-620W.7 was also seen in A375 tumors. A plausible explanation for this is the enhanced permeability retention effect, in which leaky neovasculature, combined with a lack of lymphatic drainage can result in the non-specific accumulation of high molecular weight biologics within tumors.^34^

^89^Zr-620W.7 off-target uptake was seen in both the liver and spleen, a pattern characteristic of antibody-based imaging agents owing to their interaction with Fc-γ-receptors on immune cells within these sites. Glycoengineering of antibody Fc regions has previously been explored as an effective strategy through which to diminish uptake via this mechanism.^35^ Hence, it may be possible to optimize the structure of 620W.7 to further improve its potential to act as a novel PET-imaging tracer, or alternatively as a targeted therapeutic agent as part of antibody-drug or radionuclide conjugates. Indeed, it is possible to envisage a scenario in which A20 peptides and 620W.7 could form a theranostic partnership, with the rapid trafficking of the peptide-based radiotracer facilitating diagnosis of αvβ6-expressing tumors that may be amenable to subsequent therapeutic intervention using 620W.7-targeted agents.

^89^Zr-Ad5.KO1.A20 uptake was low in both A375 and A375-β6 tumors (Figure 6C). This was despite Ad5.KO1.A20 having demonstrated a capacity to selectively bind αvβ6-expressing cell lines *in vitro* (Figures 2, S2, and S3), suggesting that its failure to traffic to A375-β6 tumor xenografts relates to suboptimal *in vivo* stability. While A20 has previously been reported to suffer from protease-mediated degradation upon exposure to serum,^36^^,^^37^^,^^38^ we did not observe a decrease in the ability of the Ad5.KO1.A20 protein to bind A375-β6 cells following its incubation in murine serum at 37°C (Figure S2). Levels of liver and renal sequestration were notably both quite high for this tracer (Figures 5E–5G), and its excretion rate was faster than for the 620W.7 and streptavidin-A20 tracers (Figure 6). It is possible that minor contributions by each of these factors combined to result in the overall lack of *in vivo* tumor accumulation seen for Ad5.KO1.A20. Alternatively, another presently unexplored clearance mechanism may underlie this observation. Greater insights into this may be gained by performing high-performance liquid chromatography on the blood and urine of ^89^Zr-Ad5.KO1.A20-injected mice to evaluate its metabolism into breakdown products and clearance kinetics.

The present study was conducted using immunodeficient mice bearing human tumor xenografts, which prevented us from evaluating the impact of interactions with the immune system on the biodistribution of the investigated agents. Additional studies confirming retention of the αvβ6-selective trafficking properties of 620W.7 and streptavidin-A20 in the presence of intact immune system would further strengthen their translational potential. Furthermore, therapeutic use of the rat monoclonal 620W.7 antibody would require its humanization such as via engraftment of its complementarity-determining regions onto a human scaffold to reduce its immunogenicity. Nevertheless, the results of this study indicate that 620W.7 and streptavidin-A20 could form the basis of highly effective αvβ6-imaging agents. Future studies could also seek to examine whether therapeutic benefit may be gained by utilizing the agents to selectively deliver therapeutic payloads to αvβ6-expressing tumors as part of targeted antibody/peptide-drug conjugates.

## Methods

### Reagents

The A20 peptide (NAVPNLRGDLQVLAQKVART) was synthesized by Peptide Synthetics Peptide Protein Research Ltd (Bishops Waltham, UK) with bi-terminal PEG-6 linkers and an N-terminal biotin modification to >95% purity. Streptavidin was purchased from Invitrogen (Rockford, Illinois, USA). The rat anti-human αvβ6 antibody clone 620W.7 was provided by John Marshall (Barts Cancer Institute, London, UK).

### Production of recombinant fiber knob proteins

Sequences encoding adenoviral fiber knob proteins were synthesized and subcloned into pQE-30 expression vectors by GENEWIZ (Leipzig, Germany). Cultures of SG13009 *E.coli* harboring the relevant plasmid were grown to an OD_600_ of 0.6, and recombinant protein expression induced via addition of isopropyl-b-d-thiogalactopyranoside to a final concentration of 0.5 mM. Culture was continued for 16 h at 20°C and 6xHis-tagged protein purified from clarified bacterial lysates under native conditions using Ni-NTA agarose resin (QIAGEN, Hilden, Germany). Protein was gradient eluted as 1 mL fractions in buffers containing 50, 100, 200, and 300 mM imidazole in addition to 50 mM Tris, 300 mM NaCl, and 1 mM β-mercaptoethanol (pH 8.0).

### (Radio) SDS-PAGE analysis

1-5 μg protein was loaded into wells of 4%–12% Bis-Tris gels (Invitrogen, Carlsbad, California, USA) and run at 150 V for 1 h 15 min. Samples prepared under denaturing conditions were boiled at 95°C in the presence of 0.05 M DTT prior to loading. Protein bands were visualized using SimplyBlue SafeStain (Invitrogen). For Radio-SDS-PAGE, ^89^Zr-labeled sample-containing lanes were excised from gels and analyzed using a Scan-RAM radio-TLC scanner (LabLogic, Sheffield, UK).

### Protein modeling

*In silico* structural predictions for recombinant adenoviral fiber knob proteins were generated using AlphaFold-Multimer run via the ColabFold notebook (v.1.5.5).^39^^,^^40^^,^^41^

### Protein sequence alignments

Protein sequence alignments were produced using the ClustalW algorithm in BioEdit (v.7.2.5).^42^

### Cell lines

All cell lines were maintained at 37°C in a humidified 5% CO_2_ atmosphere. A375 and A375-β6 cells were provided by John Marshall (Bart’s Cancer Institute, London, UK) and cultured in DMEM (Sigma-Aldrich, St. Louis, Missouri, USA), while BT-20 cells were purchased from American Type Culture Collection (ATCC, Manassas, Virginia, USA) and grown in MEM α (Sigma-Aldrich). Both cell lines were authenticated by short tandem repeat profiling conducted by Northgene (Deeside, UK). 4T1-β6 cells were generated in-house via MMLV-mediated overexpression of the murine β6 subunit in cells obtained from Awen Gallimore and Andrew Godkin (Cardiff University, UK) and grown in RPMI-1640 (Sigma-Aldrich). Culture media was supplemented with 10% FBS (20% for 4T1-β6 cells), 2% penicillin-streptomycin, and 1% L-glutamine (all Sigma-Aldrich).

### Cell surface receptor staining

2 × 10^5^ cells of the relevant cell line were incubated in 100 μL of 620W.7 (5 μg/mL), anti-αvβ6 antibody (clone 10D5, 2–5 μg/mL, [Merck Milipore, Burlington, Massachusetts, USA]), or relevant isotype controls (Rat IgG1 Kappa [RTK2071, BioLegend, Sn Diego, California, USA] or Mouse IgG2a kappa [MG2a-53, Abcam, Cambridge, UK]) on ice for 1 h, then washed twice prior to addition of 100 μL of relevant secondary antibody solution (Alexa Fluor 488 goat anti-rat [#Poly4054, BioLegend] or Alexa Fluor 647 goat anti-mouse [#A21237, Life Technologies, Eugene, Oregon, USA]). Cells were incubated with secondary antibodies in the dark for 30 min on ice, washed three times, and then fixed in 2% paraformaldehyde (Sigma-Aldrich). 10,000 single live cell events were acquired using a BD Accuri C6 plus instrument (BD Biosciences, Franklin Lakes, New Jersey, USA). Data analysis was performed using FlowJo (v.10.10.0, BD Biosciences).

### *In vitro* binding assays

10-fold serial dilutions of αvβ6-targeted agents were prepared in PBS from 0.01 to 1,000 nM and incubated with 1 × 10^5^ A375 or A375-β6 cells on ice for 30 min. Cells were washed three times, then binding assessed in flow cytometry-based assays as outlined previously. Fiber knob binding was detected using an anti-6xHisTag antibody (clone HIS.H8, 3.3 μg/mL, [antibodies.com, Cambridge, UK]), while an anti-streptavidin antibody (clone 3A20.2, 2 μg/mL, [BioLegend]) was used to assess binding of the streptavidin-A20 complex, both followed by secondary staining with Alexa Fluor 647 goat anti-mouse antibody (#A21237, Life Technologies). 620W.7 was detected using a goat anti-rat IgG Alexa Fluor 488 antibody (1 μg/mL, #Poly4054, BioLegend).

For assessment of agent stability in murine serum, samples were diluted in PBS or pooled serum from 9 to 10-week-old female CD-1 nude mice to a final concentration of 1 μM and incubated at 37°C for 0.5–48 h. Samples were diluted 10-fold in PBS prior to use in binding assays.

### ^89^Zr-radiolabelling

^89^Zr-radiolabelling was performed according to the methods of Vosjan et al.,^43^ adapted for smaller scale reactions.^44^ For streptavidin only, an additional succinylation step was performed following conjugation of the *p*-SCN-Bn-deferoxamine chelator (Macrocyclics, Plano, Texas, USA) as described previously.^33^ Mock-labeled samples were prepared by incubating DFO-conjugated proteins with ^nat^ZrCl_4_ (Sigma-Aldrich, St. Louis, Missouri, USA), prepared in 1 M oxalic acid to radiological concentrations, as opposed to [^89^Zr] during the final incubation.

### Radio-thin layer chromatography

4 μL of ^89^Zr-labeled proteins were spotted onto baselines of solid phase glass microfiber chromatography paper strips (Agilent Technologies, Santa Clara, California, USA) and allowed to air dry prior to being placed into 50 mM DTPA eluent (pH 7.4). Strips were left in the mobile phase until the solvent front reached 105 mm, then analyzed using a Scan-RAM radio-TLC scanner with Laura radiochromatography data analysis software (LabLogic).

### Longitudinal micro-PET imaging and data analysis

5–6-week-old female CD-1 nude mice were purchased from Charles River Laboratories. All experiments were conducted in accordance with the UK Home Office regulations (ASPA 1986) under project license PBEB09FBB, with local approval from the Cardiff University Animal Welfare and Ethics Committee. 2 × 10^6^ A375 or A375-β6 cells were subcutaneously injected bilaterally into the left and right hip regions of mice respectively and allowed to grow for 19 days. 2 MBq of the relevant radiotracer was then injected via the tail vein in a total volume of 20 μL (*n* = 5 or 6 mice per group). 100 μL/mouse iopamidol CT contrast agent was further administered intraperitoneally prior to imaging (Niopam 300, Bracco, Milan, Italy). 1 h Micro-PET scans were performed on 5% isoflurane in O_2_ (1.5 L/min)-anaesthetized mice placed in a prone position in a nanoScan 122S PET/CT scanner (Mediso, Budapest, Hungary) at 0.33, 24, 48, and 72 h post-radiotracer injection. An additional scan was performed after 144 h for the 620W.7 group only. PET scans were followed immediately by 3-min CT scans, and PET/CT scans reconstructed with Nucline software (v,3.04, Mediso, Budapest, Hungary) using the Tera-Tomo-3D reconstruction algorithm with 4 iterations of 6 subsets and a 0.4 mm voxel size. Attenuation and scatter corrections were applied.

Data analysis performed using VivoQuant (v.4.0 patch 3, Invicro, Needham, Massachusetts, USA). Decay corrected total radioactivity within each region was expressed as a percentage of the initial injected activity for each mouse and normalized to region of interest volume (% injected activity/mL, %IA/mL). Only mice that underwent successful radiotracer tail vein injections were included in downstream analysis. Furthermore, tumors were excluded from biodistribution analysis if these could not be reliably selected as regions of interest within overlaid PET/CT images. Some mice had to be culled prior to the end of the study owing to humane endpoints being reached. *n* numbers are indicated in figure legends.

### Calculation of radiotracer excretion rates

Radiotracer excretion rates were determined by plotting the sum of decay-corrected radioactivity contained within whole body PET scans for each mouse, expressed as a percentage of the total activity present in the first scan.

### *Ex vivo* dosimetry

Radioactivity measurements for organs and tumors harvested from mice culled following the final imaging time point were made using a sodium iodide well scintillation detector (Nuclear Enterprises, Reading, UK) and normalized to tissue mass to obtain % injected activity/g (%ID/g) values.

### Statistical analysis

Statistical analysis was performed in GraphPad prism (v.10.4.1, Dotmatics, San Diego, California, USA). Non-linear regression curves were fitted using the specific binding with Hill slope equation. Comparison of radiotracer uptake within A375 and A375-β6 tumors was performed by fitting a mixed-model with Geisser-Greenhouse correction and Šídák’s multiple comparisons post-hoc test. Statistical analysis of excretion rates was performed as described previously, with Dunnett’s post-hoc test. Comparison of uptake of the radiotracers into tumor xenografts and organs of interest across the *in vivo* imaging time course was performed by conducting area-under-curve analysis followed by Welch’s ANOVA with Dunnett’s T3 multiple comparisons test. *Ex vivo* tumor and organ dosimetry measurements were compared using Welch’s ANOVA with Dunnett’s T3 multiple comparisons test. ∗adjusted *p* value < 0.0332, ∗∗adjusted *p* value < 0.0021, ∗∗∗adjusted *p* value < 0.0002, ∗∗∗∗adjusted *p* value<0.0001, ns: no significant difference.

## Data and code availability

The datasets generated during and/or analyzed during the current study are available from the corresponding author on reasonable request.

## Acknowledgments

Schematics were produced in Biorender.com (Toronto, Ontario, Canada).

Funding was provided by a Cardiff University Innovation for All Project Grant awarded to R.J.B. and Wales Applied Virology Unit (WAVU). E.A.S. and R.J.B. were supported by a 10.13039/501100000289Cancer Research UK Biotherapeutic Program Grant awarded to A.L.P. (C52915/A29104).

The authors are grateful to Dr. Carly Bliss for providing access to CD-1 nude mouse serum, Dr. Joanna Zabkiewicz for useful discussions and Dr. Alicia Teijeira-Crespo for generation of 4T1-β6 cells.

## Author contributions

All authors contributed to the study conception and design. Material preparation, data collection, and analysis were performed by E.A.S, S.J.P., and R.J.B. Animal experiments were performed by S.J.P. and R.J.B. The first draft of the manuscript was written by E.A.S. and all authors commented on previous versions of the manuscript. All authors read and approved the final manuscript.

## Declaration of interests

A.L.P. is CSO of Trocept Therapeutics, part of Accession Therapeutics Ltd. Accession Therapeutics Ltd had no involvement in the design or execution of this study.

## Supplemental information

Document S1. Figures S1–S7

Video S1. Bladder and bowel-mediated radiotracer excretion videos Time series videos generated by dividing micro-PET scans obtained at 0.33 h into 6 × 10 min segments. High radioactivity levels are visible in the intestines and bladders of (A) ^89^Zr-Ad5.KO1.A20 and (B) ^89^Zr-Ad5.KO1-injected mice, but not equivalent regions of mice injected with (C) ^89^Zr-streptavidin-A20 or (D) ^89^Zr-620W.

Document S2. Article plus supplemental information
