# Supplementary material for: Comparison of the in vivo biodistributions of αvβ6-binding agents for PET imaging applications
Source: Mol Ther Oncol. 2026 May 14;34(2):201236. doi: 10.1016/j.omton.2026.201236 (PMC13233757; doi:10.1016/j.omton.2026.201236)
Supplement: Document S1. Figures S1–S7 [file mmc1.pdf]

OMTON, Volume *34*

Supplemental information

Comparison of the *in vivo* biodistributions of αvβ6-binding agents for PET imaging applications

Emma A. Swift, Stephen J. Paisey, Toby J. Phesse, John F. Marshall, Alan L. Parker, and Rebecca J. Bayliss

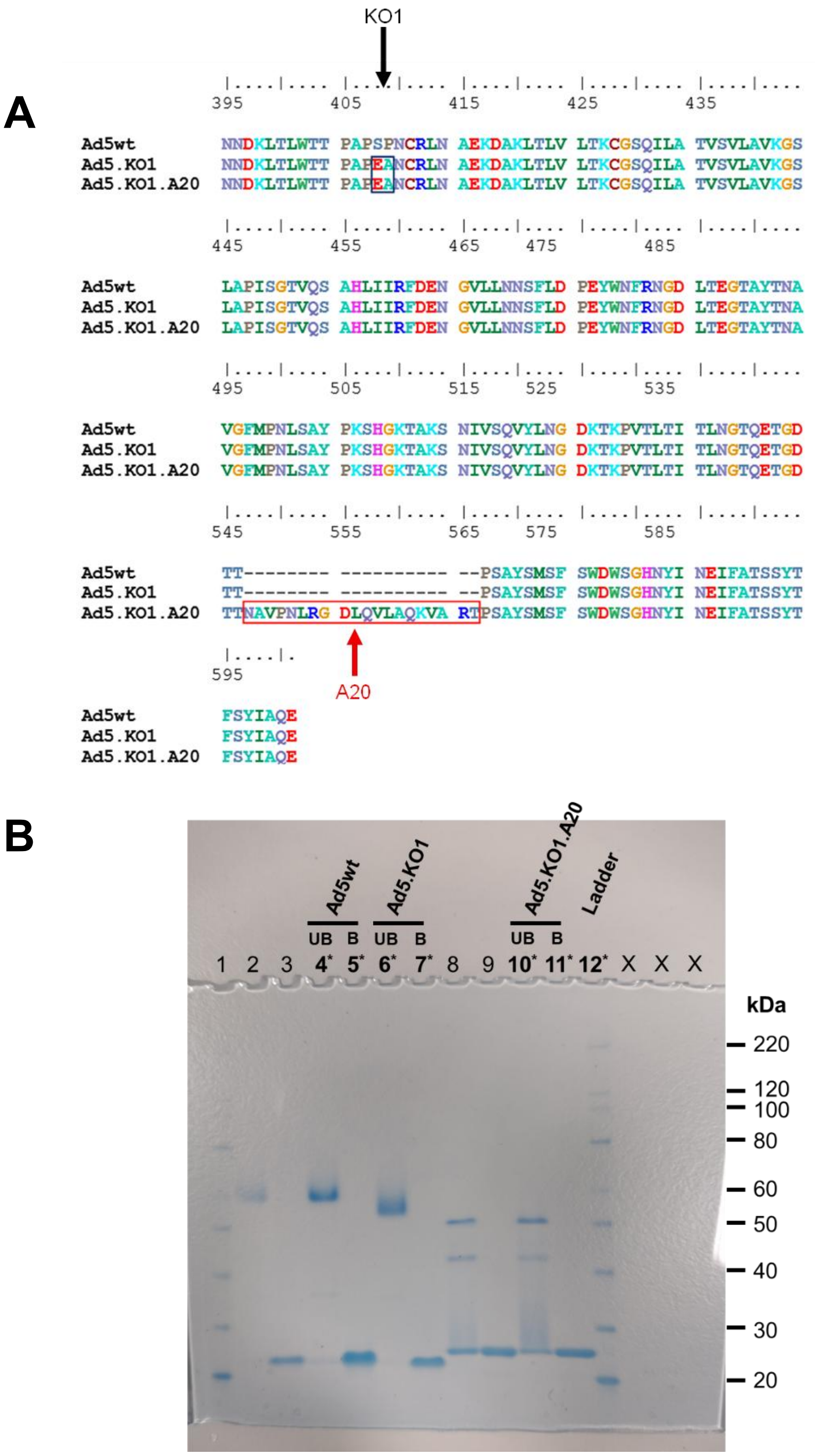

Figure S1. Fiber knob sequence alignments for the Ad5 wildtype (wt), Ad5.KO1 and Ad5.KO1.A20 fiber knob proteins. (A) The positions of the KO1 mutation (black box) and A20 peptide insert (red box) are indicated. Alignments were produced using the ClustalW algorithm in BioEdit v7.2.5. (B) Full gel image related to Figure 1C. Lanes included in Figure 1C are denoted in bold typeface with superscript asterisks. Lanes marked with an X were not loaded with sample.

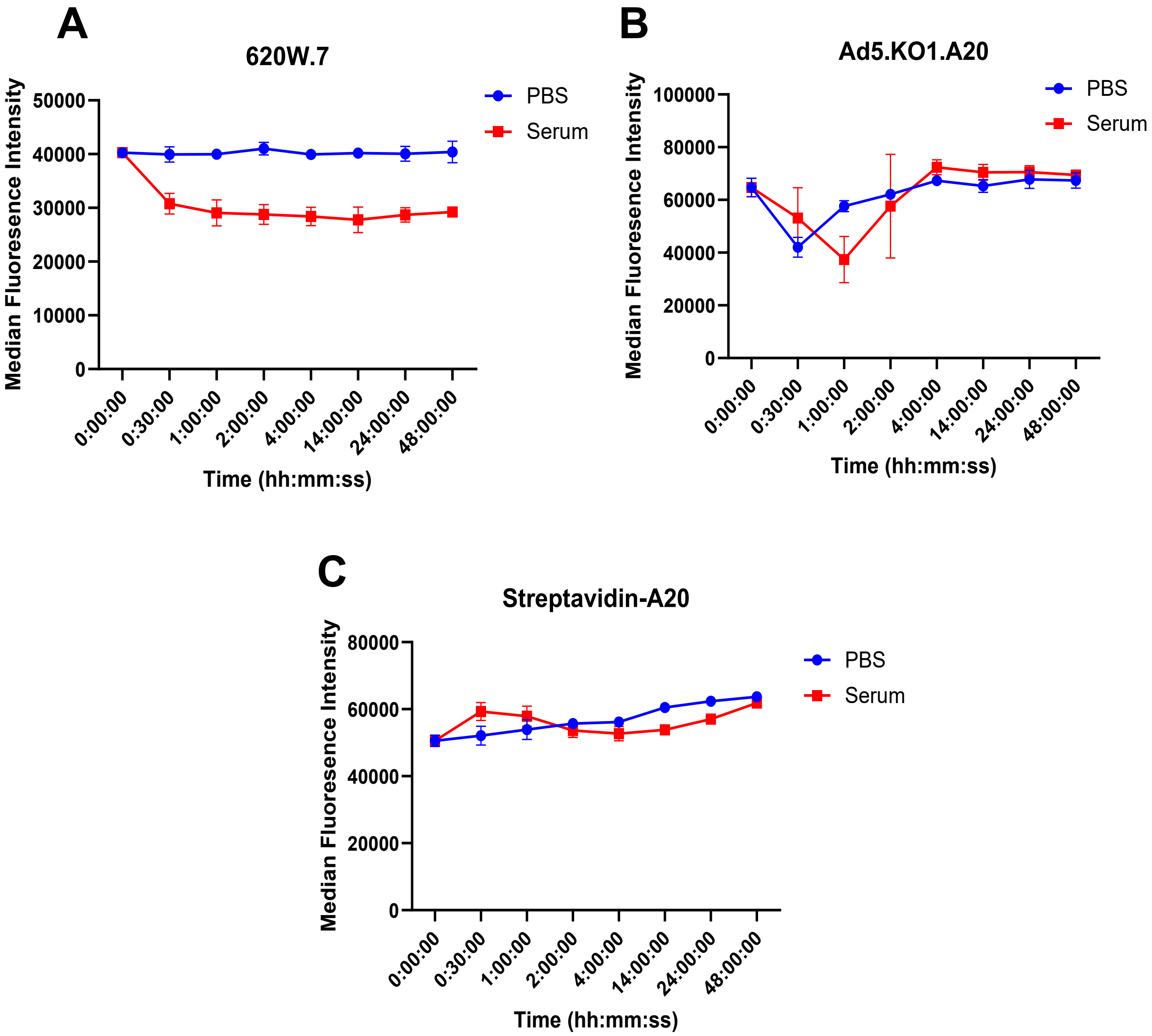

Figure S2. Serum stability of αvβ6-targeted agents. Binding of (A) 620W.7, (B) recombinant Ad5.KO1.A20 and (C) streptavidin-A20 samples to A375-β6 cells following their incubation in PBS (blue circles) or CD-1 nude mouse serum (red squares). Data are mean of three technical replicates ± standard deviation.

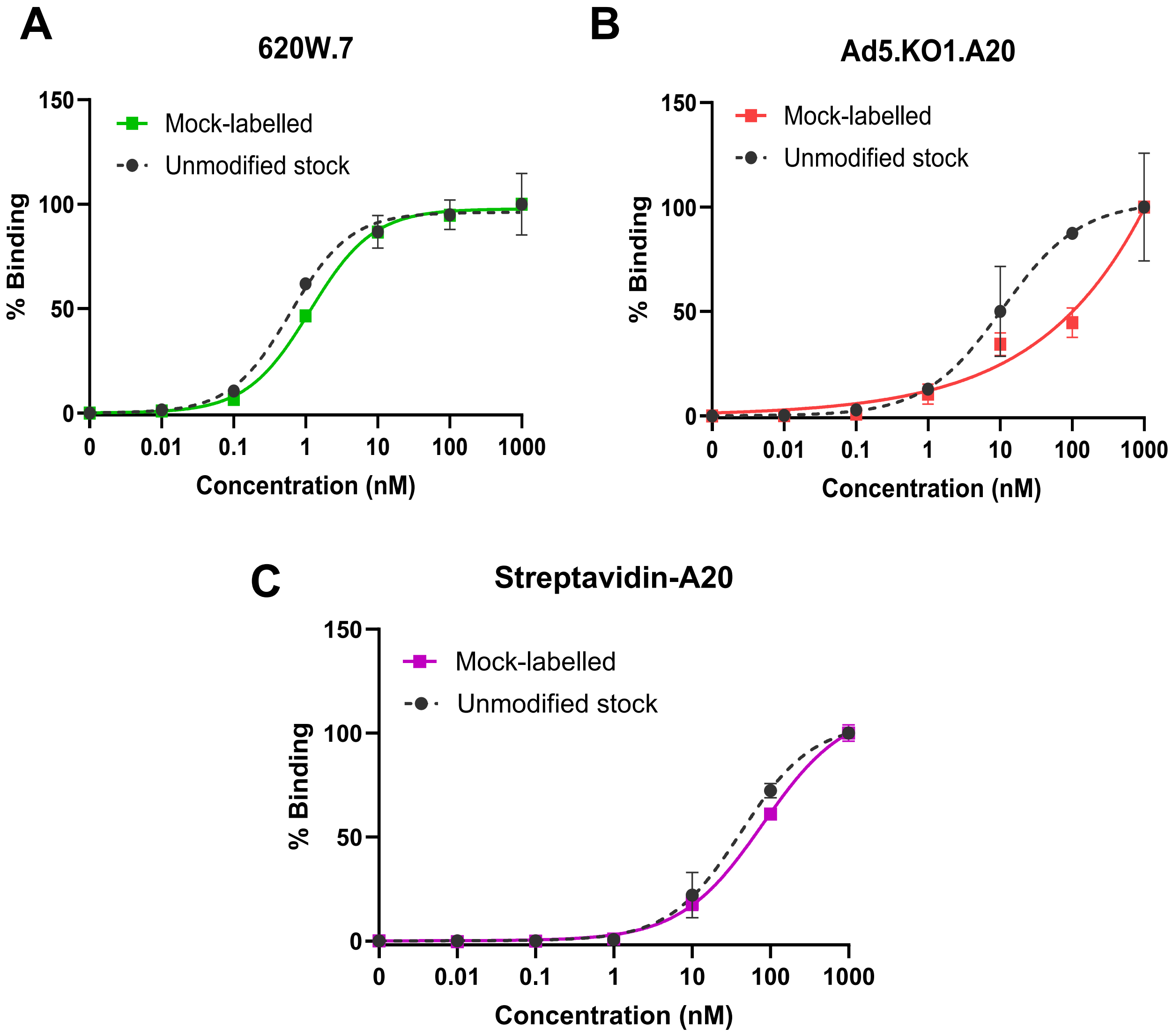

Figure S3. Comparison of unmodified and mock Zr-labelled αvβ6-targeted agent binding to A375-β6 cells. A375-β6 cells were incubated with increasing concentrations of (A) 620W.7, (B) Ad5.KO1.A20 fiber knob proteins, or (C) streptavidin-A20. Mock-labelled samples were prepared by incubating *p*-SCN-Bn-DFO conjugated samples with non-radioactive natural Zr isotopes. Mock-labelled streptavidin was subsequently incubated with biotinylated A20 in a 1:4 streptavidin:A20 molar ratio. Binding of mock-labelled samples (solid, coloured lines) was compared to unmodified stocks (dashed black lines) of each agent. 0% binding was defined as the MFI for the 0 nM condition, and 100% binding as the MFI for the 1000 nM concentration for each dataset. Data are presented as mean of 3 technical repeats ± standard deviation.

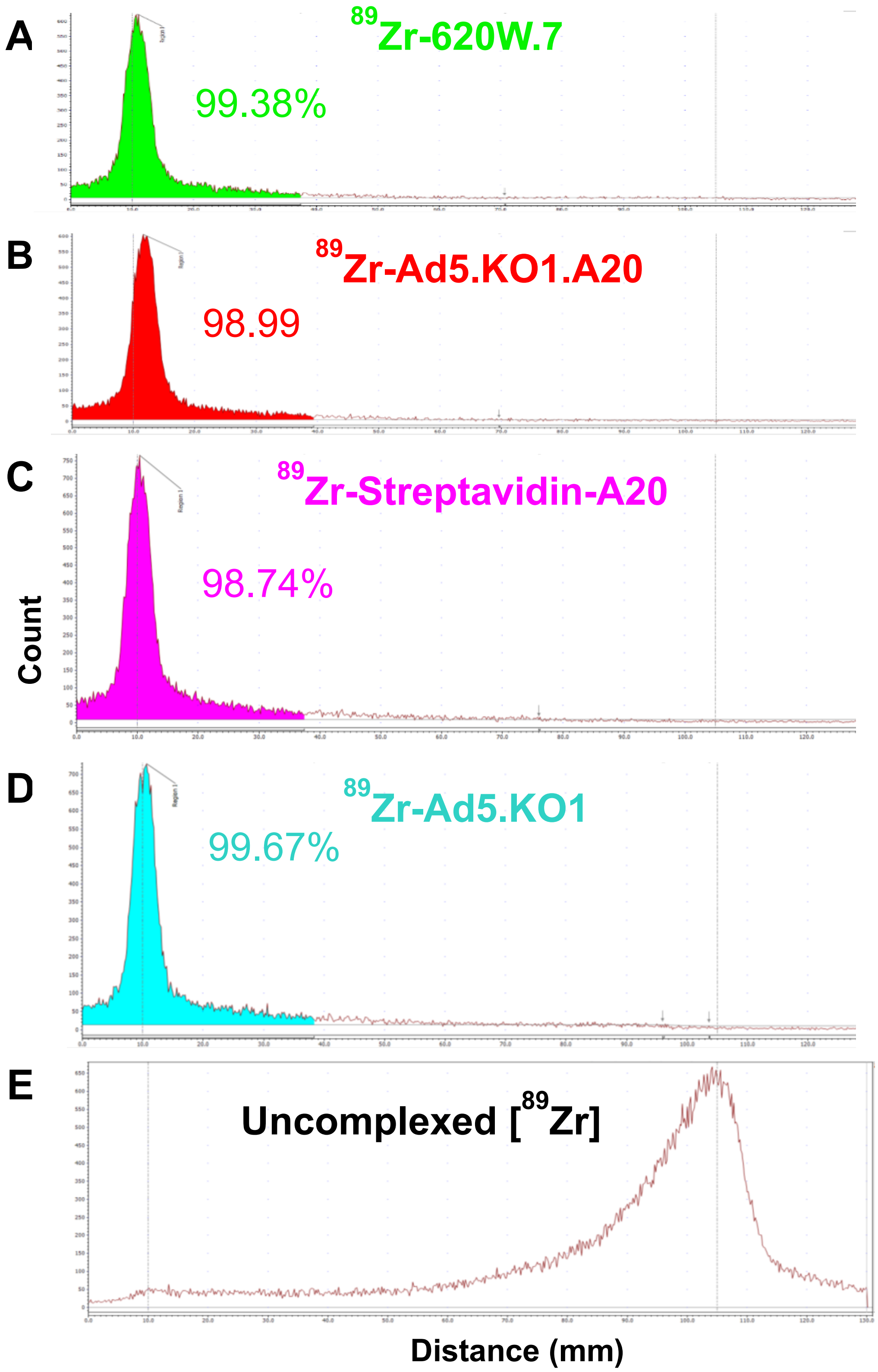

Figure S4. Evaluation of radiotracer yields by radio thin-layer chromatography. Radio-TLC chromatograms for (A) ^{89}Zr-620W.7, (B) ^{89}Zr-Ad5.KO1.A20, (C) ^{89}Zr-streptavidin-A20, and (D) ^{89}Zr-Ad5.KO1. Grey arrows indicate points on the trace selected to set the baseline. The area (% of total) contained within each of the highlighted peaks is indicated. (E) The radio-TLC trace for uncomplexed ^{89}Zr is shown for comparison.

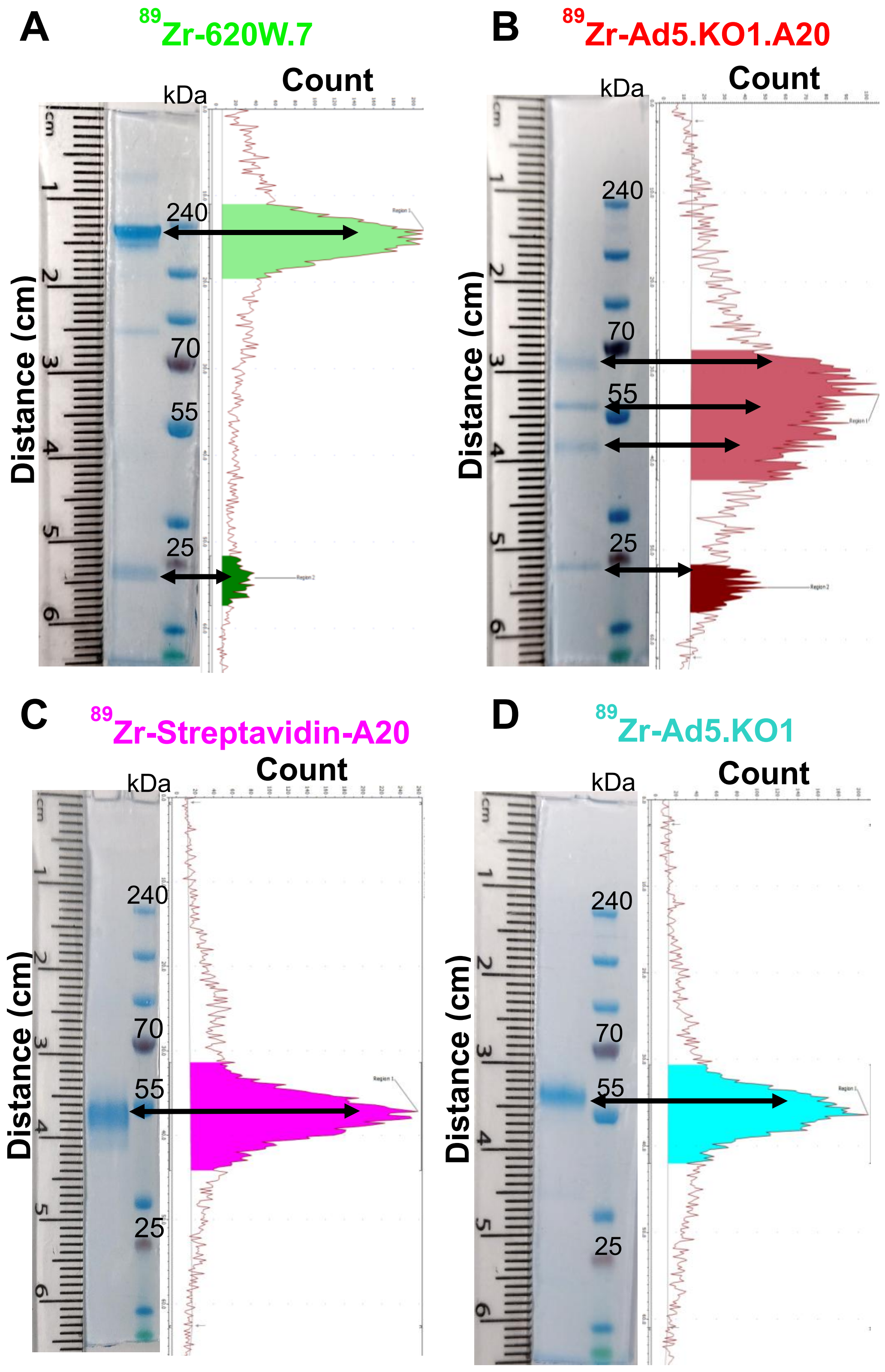

Figure S5. Assessment of radiotracer purity by radio-SDS-PAGE. Images of SDS-PAGE gels for (A) ^{89}Zr-620W.7, (B) ^{89}Zr-Ad5.KO1.A20, (C) ^{89}Zr-streptavidin-A20, and (D) ^{89}Zr-Ad5.KO1, with protein bands visualized by Coomassie blue staining, and corresponding radiochromatograms produced by analysing the relevant gel lane with a radio-TLC scanner.

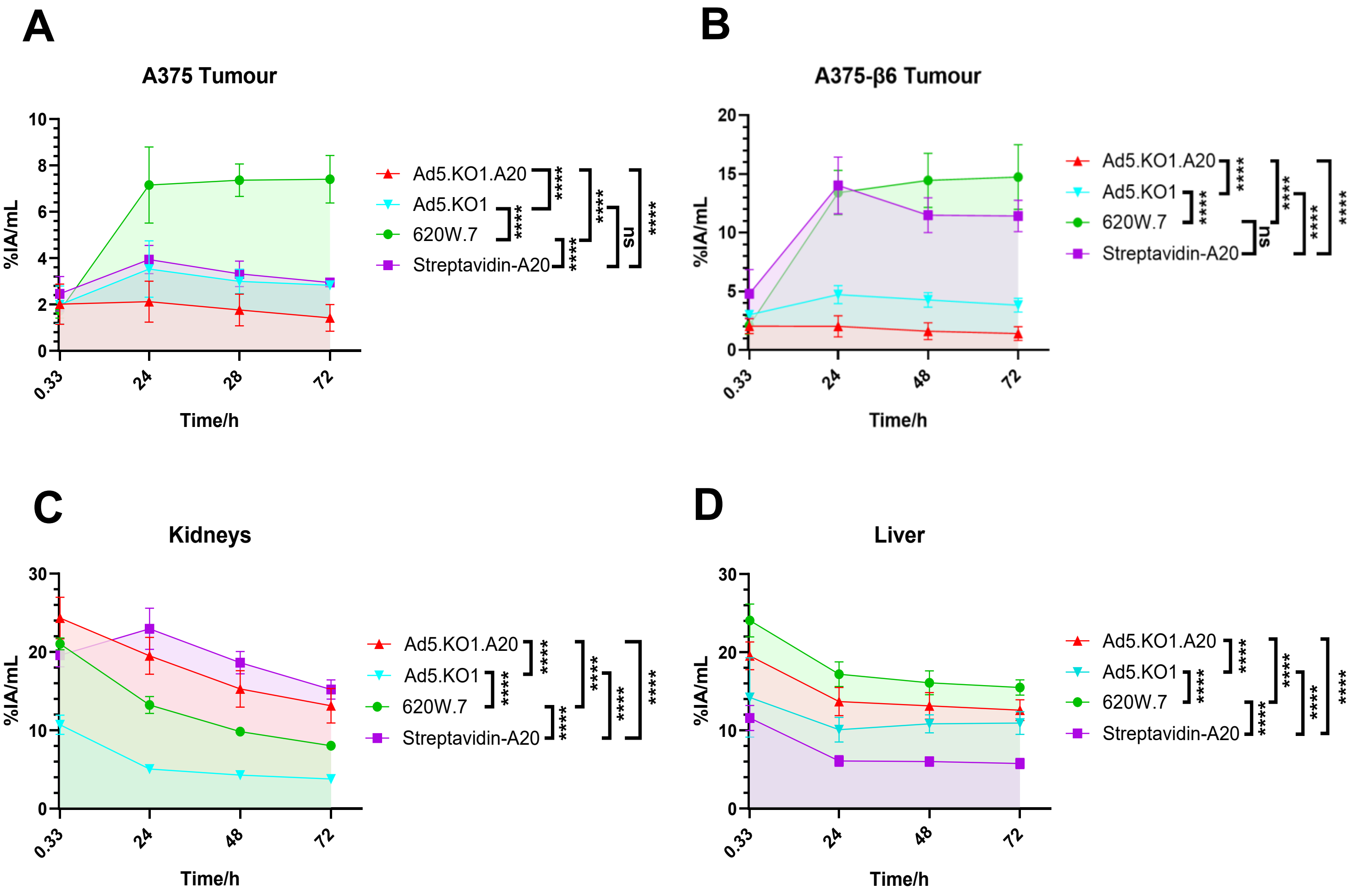

Figure S6. Comparison of the *in vivo* biodistributions of ^{89}Zr-620W.7, ^{89}Zr-Streptavidin-A20, ^{89}Zr-Ad5.KO1.A20 and ^{89}Zr-Ad5.KO1 radiotracers. Graphs depicting uptake of αvβ6-targeted agents into (A) A375 tumours (B) A375-β6 tumours (C) kidneys and (D) livers of mice injected with ^{89}Zr-labelled Ad5.KO1.A20 (red triangles), Ad5.KO1 (blue inverted triangles), 620W.7 (green circles) or streptavidin-A20 (purple squares) across the 0.33-72 h imaging timepoints. N numbers as in main text figures 5E (Ad5.KO1.A20), 5F (Ad5.KO1), 4D (620W.7), and 3D (streptavidin-A20). Data are presented as mean ± standard deviation. Statistical analysis was performed by comparing area-under-curve for each agent using Welch's ANOVA with Dunnett's T3 multiple comparisons test. ****adjusted $p<0.0001$, ns: no significant difference.

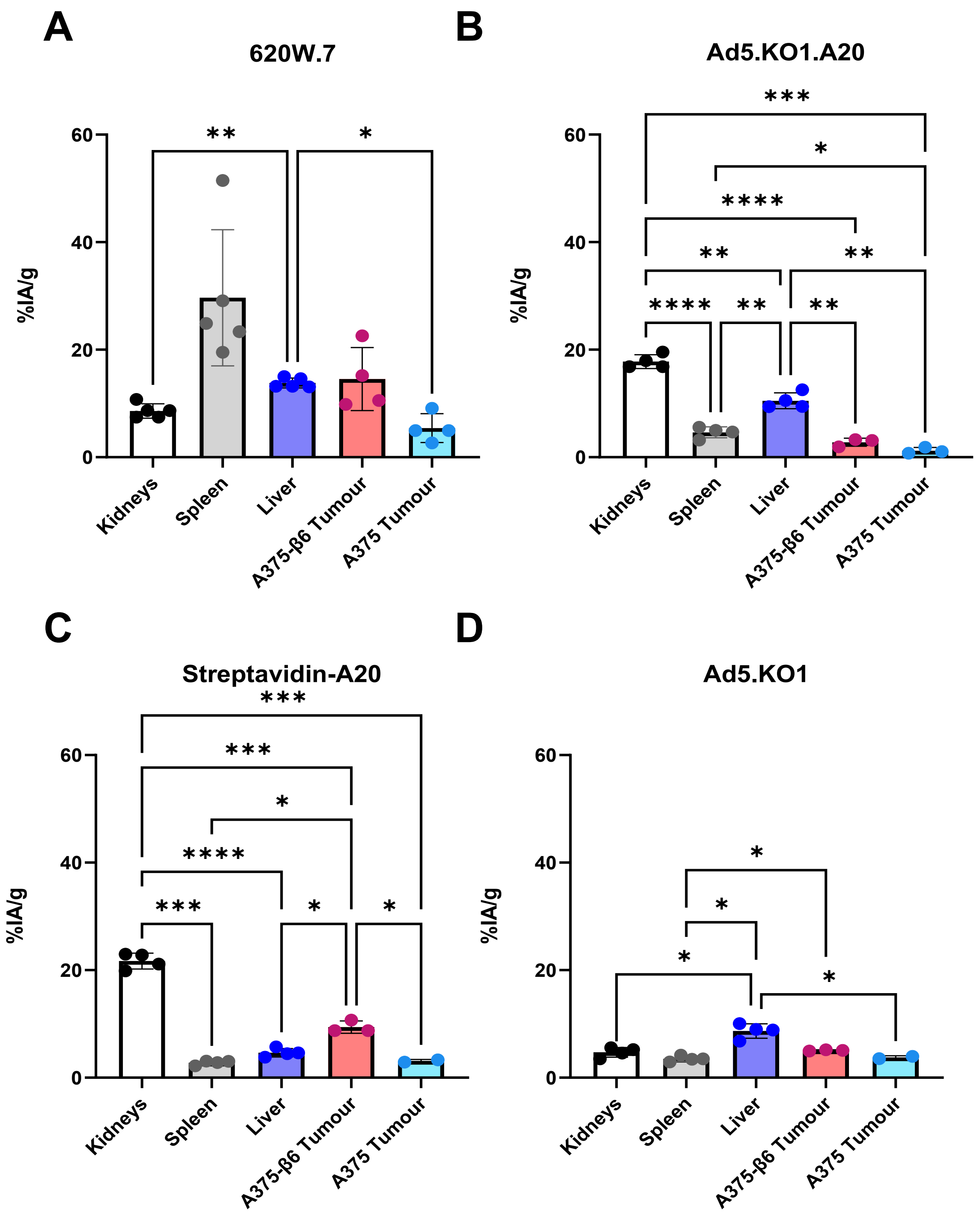

Figure S7. ***Ex vivo*** **biodistribution analysis.** Radioactivity contained within organs and tumours harvested from mice injected with (A) ^{89}Zr-620W.7 (n=5 for organs, n=4 for tumours), (B) ^{89}Zr-Ad5.KO1.A20 (n=4 for organs, n=3 for tumours), (C) ^{89}Zr-streptavidin-A20 (n=4 for organs, n=3 for A375-β6 tumours, n=2 for A375 tumours) and (D) ^{89}Zr-Ad5.KO1 (n=4 for organs, n=3 for A375-β6 tumours, n=2 for A375 tumours) following the final imaging timepoint for each agent (144 h post-injection for 620W.7, 72 h post-injection for the remaining agents). Data are presented as mean ± standard deviation. Statistical analysis was performed using Welch's ANOVA with Dunnett's T3 multiple comparisons test. *adjusted p<0.0332, **adjusted p<0.0021, ***adjusted p<0.0002, ****adjusted p<0.0001.

Video S1. Bladder and bowel-mediated radiotracer excretion videos. Time series videos generated by dividing micro-PET scans obtained at 0.33 h into 6x10 minute segments. High radioactivity levels are visible in the intestines and bladders of (A) ^{89}Zr-Ad5.KO1.A20 and (B) ^{89}Zr-Ad5.KO1-injected mice, but not equivalent regions of mice injected with (C) ^{89}Zr-streptavidin-A20 or (D) ^{89}Zr-620W.
