# Supplementary material for: Comparison of the in vivo biodistributions of αvβ6-binding agents for PET imaging applications
Source: Mol Ther Oncol. 2026 May 14;34(2):201236. doi: 10.1016/j.omton.2026.201236 (PMC13233757; doi:10.1016/j.omton.2026.201236)
Supplement: Document S2. Article plus supplemental information [file mmc3.pdf]

Molecular Therapy
Oncology
Original Article

Comparison of the *in vivo* biodistributions of αvβ6-binding agents for PET imaging applications

Emma A. Swift,[1] Stephen J. Paisey,[2] Toby J. Phesse,[3,4] John F. Marshall,[5] Alan L. Parker,[1,6] and Rebecca J. Bayliss[1,6]

[1]Division of Cancer and Genetics, School of Medicine, Cardiff University, Cardiff CF14 4XN, UK; [2]Wales Research and Diagnostic Positron Emission Tomography Imaging Centre, School of Medicine, Cardiff University, Cardiff CF14 4XN, UK; [3]European Cancer Stem Cell Research Institute, School of Biosciences, Cardiff University, Cardiff CF24 4HQ, UK; [4]The Peter Doherty Institute for Infection and Immunity, Melbourne, VIC 3000, Australia; [5]Centre for Tumor Biology, Barts Cancer Institute, Queen Mary University of London, London EC1M 6BQ, UK; [6]Wales Applied Virology Unit (WAVU), Cardiff University School of Medicine, Heath Park, Cardiff CF14 4XN, UK

Expression of the αvβ6 integrin is upregulated on multiple solid epithelial tumors while this integrin is largely absent on the healthy human epithelium. In the present study, we sought to compare the *in vivo* biodistributions of three αvβ6-selective agents that could be used for the positron emission tomography (PET)-based detection of malignancies expressing this integrin. A streptavidin-coupled αvβ6-binding peptide (streptavidin-A20), an anti-αvβ6 monoclonal antibody (620W.7) and an αvβ6-targeted protein, derived from a modified adenovirus 5 receptor-binding domain (Ad5.KO1.A20), were radiolabeled with [^{89}Zr] and intravenously injected into mice bearing bilateral αvβ6-positive and -negative melanoma xenografts. Micro-PET imaging was performed at 0.33, 24, 48, 72, and (620W.7 group only) 144 h post-injection. Streptavidin-A20-based radiotracers preferentially accumulated in αvβ6-positive tumors, achieving >3.4:1 A375-β6:A375 activity ratios between 24 and 72 h post-injection, with off-target uptake restricted to the kidneys. 620W.7 exhibited roughly 2-fold greater accumulation in A375-β6 than A375 tumors, with lower levels of renal retention and greater activity detected in the liver and spleen. Despite having demonstrated selective binding to αvβ6-expressing cells *in vitro*, the Ad5.KO1.A20 protein failed to selectively traffic to tumors expressing this integrin *in vivo*. Nevertheless, streptavidin-A20 and 620W.7 demonstrated promise as αvβ6-selective agents for *in vivo* applications.

INTRODUCTION

Integrins are a family of non-covalently associated, heterodimeric adhesion proteins that mediate crosstalk between cells and extracellular matrix (ECM) components. αvβ6 is an epithelial-restricted member of the integrin family,[1] whose native ligands encompass several ECM glycoproteins,[2] as well as latency-associated peptides linked to transforming growth factor (TGF)-β1 and TGF-β3 molecules in large latent complexes.[3,4]

Studies probing adult tissues for β6 expression have noted its conspicuous absence from the surface of most healthy epithelia. In contrast, β6 staining is detectable within the kidneys, lungs, and skin of fetal tissues, suggesting a physiological role for αvβ6 in organogenesis, with subsequent downregulation of integrin expression following tissue differentiation.[1] However, *de novo* expression of αvβ6 has been reported to occur in epithelial carcinomas arising in several different anatomical locations, including those affecting the oral squamous epithelium,[5] pancreas,[6] colon,[7] stomach,[8] ovaries,[9] and bile duct.[10] Integrin upregulation in these settings is generally associated with poorer outcomes.[7–10]

Several therapies seeking to exploit the cancer-associated expression profile of αvβ6 have emerged in recent years, including αvβ6-targeted CAR T cells,[11,12] oncolytic virotherapy platforms,[13,14] and immunoliposomes loaded with cytotoxic cargoes.[15] The greatest responses to these therapeutics can be expected to occur in patients whose tumors express high levels of αvβ6. Molecularly targeted PET imaging offers a non-invasive means through which individuals may be stratified for treatment with such αvβ6-selective therapeutics.

PET-based detection of αvβ6-expressing malignancies has previously been facilitated through the use of radiolabeled integrin-binding peptides. This includes those based on the linear A20 peptide sequence (NAVPNLRGDLQVLAQKVART) derived from the αvβ6-engaging VP1 protein of the foot-and-mouth disease virus,[16,17] as well as peptides selected from sunflower trypsin inhibitor-1 scaffold-guided phage display libraries,[18,19] knottins,[20] and head-to-tail cyclized peptides.[21] These agents have shown promise in their ability to detect αvβ6-expressing pathologies in preclinical studies and small first in-human clinical trials.[16–24] Nevertheless, peptide tracers suffer from a propensity to be reabsorbed within the kidneys, resulting in dose-limiting nephrotoxicities.[25] Furthermore, several of the

Received 13 January 2026; accepted 11 May 2026;
https://doi.org/10.1016/j.omton.2026.201236.

Correspondence: Rebecca J. Bayliss, Cancer and Genetics Building, Heath Park Way, Cardiff CF14 4AW, UK.
E-mail: baylissr4@cardiff.ac.uk

© 2026 The Author(s).
Published by Elsevier Inc. on behalf of The American Society of Gene and Cell Therapy.
This is an open access article under the CC BY license (http://creativecommons.org/licenses/by/4.0/).

non-backbone cyclized αvβ6-binding peptide variants examined to date have been shown to accumulate in the GI tract,[18,20,22–24] limiting their capacity to accurately delineate tumors arising within this region.[26]

In the present study, we sought to compare the *in vivo* biodistribution of a streptavidin-coupled A20 peptide-based radiotracer (streptavidin-A20) with the uptake profiles of two other classes of αvβ6-targeted biologics. These consisted of a monoclonal anti-αvβ6 antibody (620W.7) and a protein derived from the primary receptor-binding domain of an αvβ6-selective adenovirus 5 virotherapy platform.[14] All three agents were labeled with the long half-life zirconium-89 [^{89}Zr] radionuclide and injected intravenously into mice bearing bilateral αvβ6-positive and -negative melanoma tumor xenografts. This facilitated evaluation of their uptake kinetics and biodistribution profiles in a longitudinal microPET imaging study.

RESULTS

Overview of αvβ6-targeted biologics

Previous investigations into the αvβ6-binding A20 peptide have revealed that its retention within tumors and *in vivo* pharmacokinetic profile can be improved by the addition of polyethylene glycol (PEG) moieties at both its N and C terminus.[27] The A20 peptide utilized in the present study was therefore synthesized with biterminal PEG-6 linkers. Furthermore, we incorporated an N-terminal biotin modification into our peptide-based radiotracer design to facilitate A20 coupling to succinylated ^{89}Zr-streptavidin in an indirect radiolabeling approach (Figure 1A). This was adopted to minimize levels of renal retention by increasing the overall molecular weight of the radiotracer. When mixed in a 4:1 peptide: streptavidin molar ratio, the predicted size of the resulting complex was 64.9 kDa.

Secondly, we generated an αvβ6-targeted protein derived from the receptor-binding domain of a modified adenovirus 5 virotherapy platform. The oncolytic Ad5$_{\mathrm{NULL}}$-A20 virus has been engineered to selectively infect and lyse αvβ6-expressing tumors.[14] Its αvβ6-dependent mechanism of cell entry is dependent upon the A20 peptide, which is inserted into a permissive loop of the virus fiber knob domain (Figure 1B). When combined with two point mutations (S408E, P409A, collectively referred to as KO1) that ablate the high affinity native interaction of this domain with the coxsackie and adenovirus receptor (CAR) (Figure S1A),[28] this results in αvβ6 integrin-dependent virus entry.[14,29] We hypothesized that the knob domain of this virus could serve as a useful αvβ6-binding agent when expressed independently of the whole viral vector. As such, we generated recombinant versions of this protein (referred to as Ad5.KO1.A20), alongside an untargeted control domain lacking the A20 insert (Ad5.KO1). Both proteins retained the native capacity of adenoviral fiber knob proteins to trimerise (Figure 1C), providing three available αvβ6-binding sites per Ad5.KO1.A20 trimer.

The final class of αvβ6-binding biologic examined in the present study consisted of an anti-αvβ6 monoclonal antibody (clone 620W.7). 620W.7 recognizes the human αvβ6 integrin heterodimer but lacks cross-reactivity with the equivalent murine isoform (Figure 1D).

In vitro evaluation of targeted agent binding to αvβ6

Serial dilutions of each of the candidate αvβ6-targeted agents were incubated with A375 cells, which lack αvβ6 but reportedly express other related members of the integrin heterodimer family,[30,31] as well as a matched A375-β6 line engineered to overexpress the human αvβ6 isoform (Figure 2A).

All three targeted agents demonstrated clear binding to the A375-β6 cell line at nanomolar concentrations without detectable binding to A375 cells, consistent with their selective, high affinity recognition of αvβ6 (Figures 2B–2D). As expected, the untargeted Ad5.KO1 protein didn't bind to either cell line (Figure 2E). Furthermore, the αvβ6-binding capacity of these agents was not unduly disrupted by either exposure to murine serum (Figure S2) or conjugation to the deferoxamine chelator required for their subsequent ^{89}Zr-radiolabelling (Figure S3).

In vivo biodistribution of αvβ6-targeted agents

The three αvβ6-targeted agents and untargeted Ad5.KO1 control protein were labeled with [^{89}Zr] to high radiochemical yields and purities as assessed by radio-TLC (Figure S4) and radio-SDS-PAGE (Figure S5). 2 MBq of each tracer was subsequently injected into the tail veins of CD-1 nude mice bearing A375 and A375-β6 tumors over their left and right hip regions, respectively.

^{89}Zr-streptavidin-A20

^{89}Zr-streptavidin-A20 radiotracers demonstrated excellent selectivity in trafficking to αvβ6-positive tumors while minimally accumulating in A375 xenografts. Across the 24–72 h imaging window, radioactivity in A375-β6 tumors remained >3.4 times that in αvβ6-negative A375 tumors, peaking at 14.03 ± 2.42% IA/mL after 24 h (Figures 3A–3C).

The major site of off-target uptake for ^{89}Zr-streptavidin-A20 was within the kidneys. Activity within this site peaked at 22.96 ± 2.62% IA/mL 24 h post-injection, before gradually declining to 15.22 ± 1.24% IA/mL by the final imaging time point. Comparatively little radioactivity was detected within the livers of these mice, remaining around 6% IA/mL, with no evidence of tracer accumulation in the GI tract or spleen (Figures 3A and 3D).

^{89}Zr-620W.7

Similar to ^{89}Zr-Streptavidin-A20 tracers, ^{89}Zr-620W.7 preferentially accumulated in A375-β6 tumors over A375 xenografts (Figures 4A–4C). Indeed, %IA/mL values for ^{89}Zr-620W.7 in A375-β6 tumors were slightly higher than those measured for the A20 peptide-based radiotracer at the 48 h and 72 h time points (Figure S6), peaking 72 h post-injection at 14.75 ± 2.75% IA/mL. However, uptake in the A375 tumors was also increased for ^{89}Zr-620W.7, resulting in only approximately 2:1 A375-β6:A375 tumor activity ratios (Figure 4C).

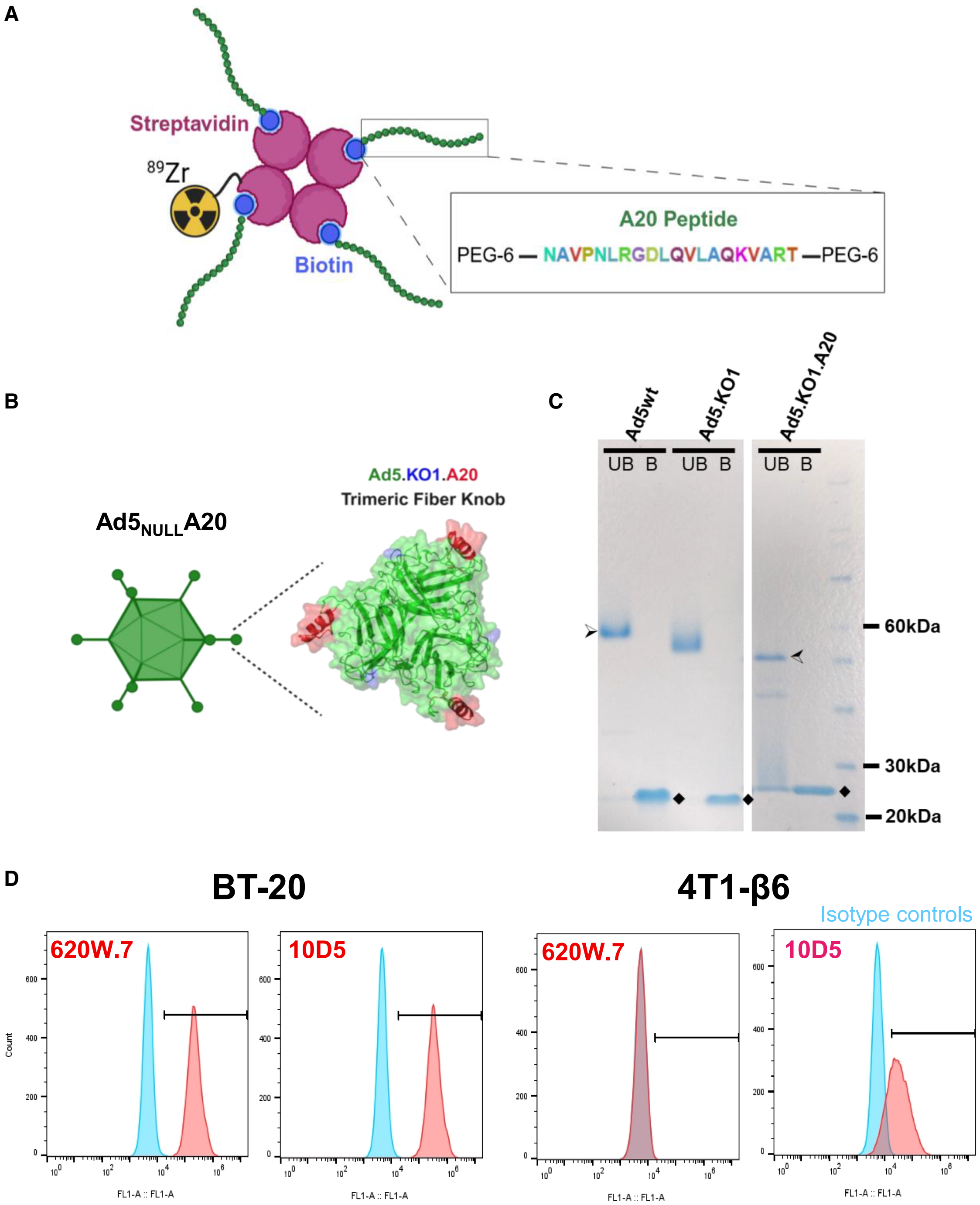

Figure 1. Overview of αvβ6-targeted biologics

(A) Schematic overview of the indirect radiolabeling approach adopted for the A20 peptide-based tracer. (B) Cartoon depicting the location of the fiber knob domain within $Ad5_{NULL}$-A20 virions (left), alongside a model for the trimeric Ad5.KO1.A20 protein. The A20 peptide insert is depicted in red, and S408E, P409A substitution mutations that ablate CAR binding (collectively referred to as KO1) are shown in blue. (C) SDS-PAGE assessment of recombinant fiber knob trimerization. Half-filled arrowheads denote bands corresponding to trimeric forms of the relevant fiber knob, while monomers are indicated by black diamonds. UB, un-boiled; B, boiled; wt, wildtype; PEG, polyethylene glycol. The full gel image is provided in Figure S1B. (D) Representative flow cytometry histograms depicting staining of BT-20 cells (which express human αvβ6) and 4T1-β6 cells (expressing the equivalent murine isoform) with the 620W.7 antibody. A second commercially available isoform cross-reactive anti-αvβ6 antibody (clone 10D5) was used as a control.

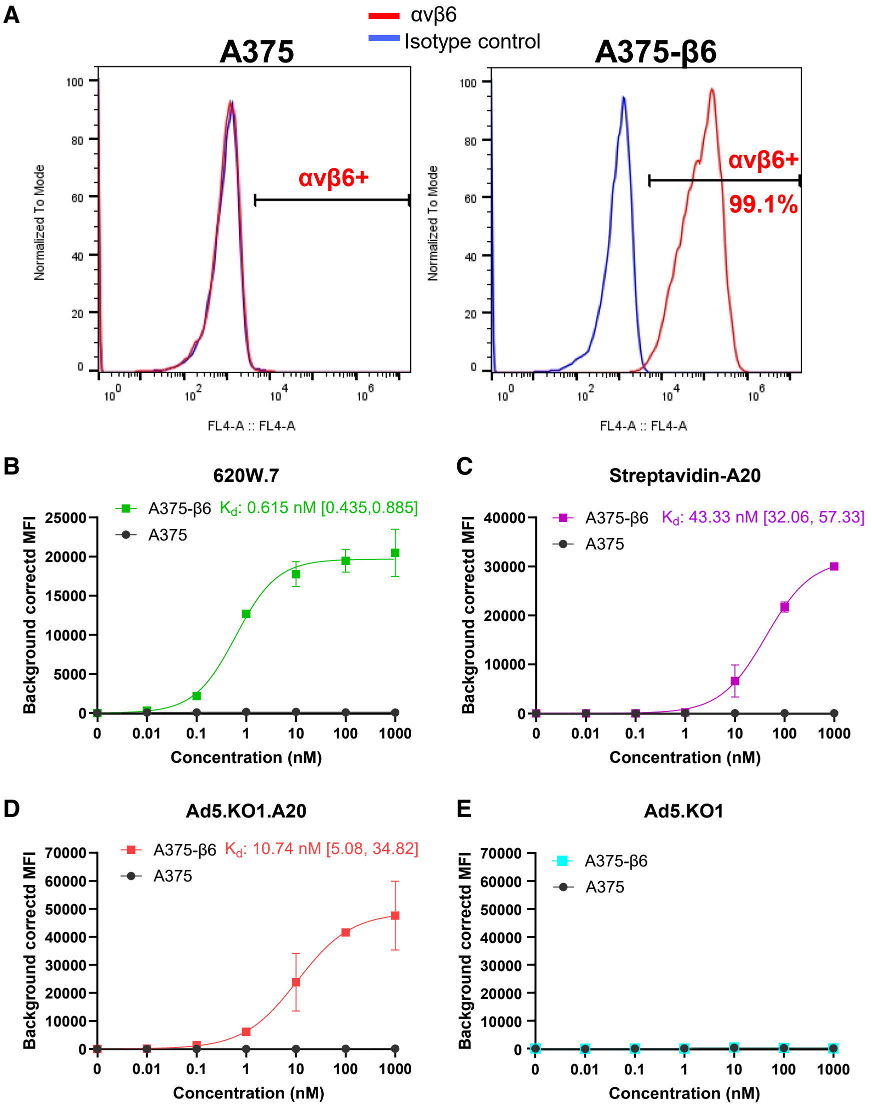

Figure 2. *In vitro* evaluation of αvβ6-targeted agent binding to A375 and A375-β6 cells

(A) Representative flow cytometry histograms depicting staining of A375 and A375-β6 cells for αvβ6. Increasing concentrations of the (B) 620W.7 antibody, (C) streptavidin-A20 complex (prepared in a 1:1 streptavidin:A20 molar ratio), (D) Ad5.KO1.A20 knob protein, and (E) Ad5.KO1 recombinant fiber knob were incubated with A375 and A375-β6 cells. Median fluorescence intensity values (MFI) were determined in triplicate for each concentration, and data background corrected to the MFI of the 0 nM condition. Non-linear regression curves were fitted using the specific binding with Hill slope equation in GraphPad Prism (v.10.4.1) and Kd values are reported alongside their 95% confidence intervals (profile likelihood). Data are presented as mean ± standard deviation.

Though initially high, radioactivity levels in the kidneys of ^{89}Zr-620W.7-injected mice dropped rapidly following the first scan, suggestive of radiotracer washout from this site. In contrast, roughly 15%–17.2% IA/mL was retained within the livers of these mice from 24 to 144 h. Similar levels of activity were also detected in their spleens (Figure 4D), with further focal accumulation of ^{89}Zr-620W.7 visible within the shoulder regions of mice, thought to correspond to uptake into axillary lymph nodes (Figures 5A and 5B).

^{89}Zr-Ad5.KO1.A20

In contrast to the other two αvβ6-targeted agents, ^{89}Zr-Ad5.-KO1.A20 radiotracers did not traffic to αvβ6-expressing tumors. %IA/mL values were <2.5% for this tracer in both A375 and A375-β6 tumors throughout the study, similar to the untargeted Ad5.KO1 control protein (Figures 6A–6D).

Radioactivity was detected in both the liver (∼13% IA/mL for scans at 24–72 h) and kidneys (starting at 24.32 ± 2.65% IA/mL, decreasing to 13.13 ± 2.21% IA/mL by 72 h) of ^{89}Zr-Ad5.KO1.A20-injected mice (Figure 5E). Interestingly, while levels of hepatic retention were similar between the ^{89}Zr-Ad5.KO1 and ^{89}Zr-Ad5.KO1.A20 groups (Figure 6G), the αvβ6-targeted protein exhibited significantly greater kidney uptake (Figure 6H).

Ex vivo dosimetry measurements corresponded well to uptake values calculated from PET imaging scans (Figure S7).

Excretion rates

High levels of radioactivity were observed within the intestines and bladders of knob protein-injected mice during initial microPET scans, whereas this was not a feature of the other two αvβ6-targeted agents (Video S1). We therefore examined whether radiotracer excretion via these routes might underpin the failure of ^{89}Zr-Ad5.KO1.A20 to traffic to αvβ6-expressing tumors *in vivo*.

While total radioactivity within scans of Ad5.KO1- and Ad5.KO1.A20-injected mice decreased faster than for the other two αvβ6-targeted agents, 46.52 ± 3.35% of the initial dose remained by the final time point (Figure 7). Hence, excretion of the Ad5.KO1.A20-based radiotracer alone cannot wholly account for its lack of uptake into A375-β6 tumors.

DISCUSSION

The contribution of αvβ6 upregulation to the progression and metastasis of several aggressive epithelial carcinomas has resulted

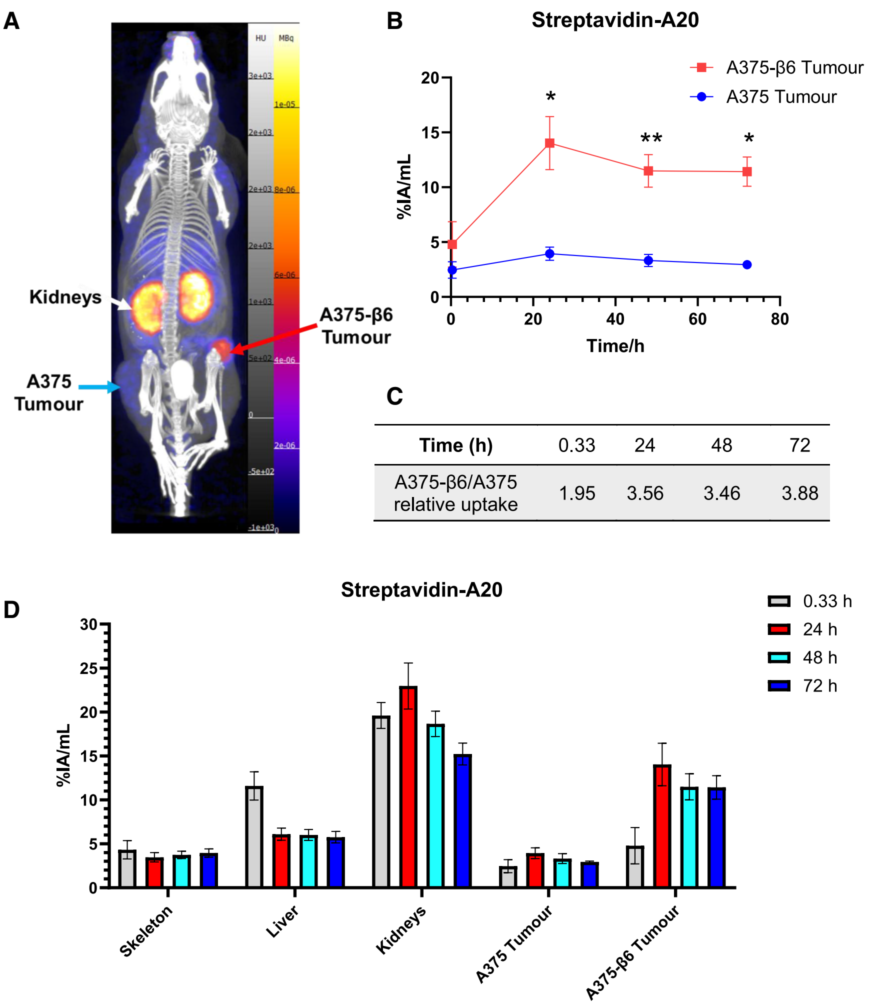

Time (h)	0.33	24	48	72
A375-β6/A375 relative uptake	1.95	3.56	3.46	3.88

Figure 3. *In vivo* biodistribution of ^{89}Zr-streptavidin-A20 radiotracers

(A) Representative overlaid maximum intensity projection (MIP) views of PET/CT scans for a mouse injected with ^{89}Zr-streptavidin-A20, obtained after 24 h. (B) Comparison of radioactivity levels in A375-β6 and A375 tumors for ^{89}Zr-streptavidin-A20-injected mice (0.33–48 h: *n* = 4 for both groups, 72 h: *n* = 3 for A375-β6 and *n* = 2 for A375 tumor groups). (C) Relative uptake in A375-β6 tumors over A375 tumors across the four imaging time points. (D) Radiotracer uptake in organs of interest for ^{89}Zr-streptavidin-A20 injected mice (tumor n numbers as in (B), all other regions 0.33–48 h: *n* = 6, 72 h: *n* = 4). Data are presented as mean ± standard deviation. *adjusted *p* value<0.0332, ** adjusted *p* value<0.0021.

in significant interest in the potential to exploit its expression as a tumor-specific vulnerability in anti-cancer therapeutic approaches.[7–10] The present study outlines the evaluation of three αvβ6-binding biologics that may be used as molecularly targeted agents for the non-invasive diagnosis and monitoring of tumors expressing this integrin.

Our findings solidify the promise of A20 peptide-based radiotracers to serve as the basis for effective αvβ6-targeted PET imaging tracers. Consistent with previous studies,[16,22,23] the A20 peptide-based radiotracer demonstrated an extremely promising *in vivo* biodistribution, rapidly accumulating in αvβ6-expressing tumors and exhibiting >3.4-fold greater accumulation in these compared to αvβ6-negative tumors over a 3-day imaging period (Figure 3).

The kidneys were the primary site of streptavidin-A20 off-target uptake (Figure 3D). Significantly greater renal retention was also observed when comparing the biodistribution of the Ad5.KO1.A20 fiber knob to the untargeted Ad5.KO1 control, which was not observed in the liver (Figures 6G and 6H). Endogenous expression of αvβ6 in murine kidneys has been shown to be negligible, while A20 peptide trapping within this site has previously been prevented by co-injection of a scrambled version of this sequence.[16,32] This suggests that renal uptake of A20-containing tracers is unlikely to occur via target-specific mechanisms and instead relates to its physicochemical properties. Notably, the levels of ^{89}Zr-streptavidin-A20 renal trapping observed here do compare favorably to those reported previously for A20-based radiotracers, particularly those making use of similar bi-terminally PEGylated peptide variants.[16,27] This may be reflective of the increased molecular weight of the tracer used here, achieved via peptide complexation to radiolabeled streptavidin, which was further succinylated to reduce its isoelectric point and propensity to be reabsorbed by proximal tubular cells.[33] Additional studies are required to determine the half-life and other specific pharmacokinetic parameters for the streptavidin-A20 tracer developed here and compare these values to existing αvβ6-binding peptides. Should our indirect labeling method prove to provide favorable pharmacokinetic properties, this could be a broadly applicable strategy for reducing the nephrotoxicity risk associated with the use of low molecular weight peptides as radiopharmaceuticals.[25] Indeed, this strategy, based entirely on commercially available agents, has the potential to work for any biotinylated peptide and could provide an alternative to antibody labeling strategies when antibodies are not available or targets in the liver or spleen are of interest.

The anti-αvβ6 monoclonal antibody 620W.7 was also effective in delineating A375-β6 tumors in our preclinical imaging study, with maximal accumulation after 72 h (Figure 4). However, while ∼2-fold lower than the activity level in matched A375-β6 tumors, weak uptake of ^{89}Zr-620W.7 was also seen in A375 tumors. A plausible explanation for this is the enhanced permeability retention effect, in which leaky neovasculature, combined with a lack of

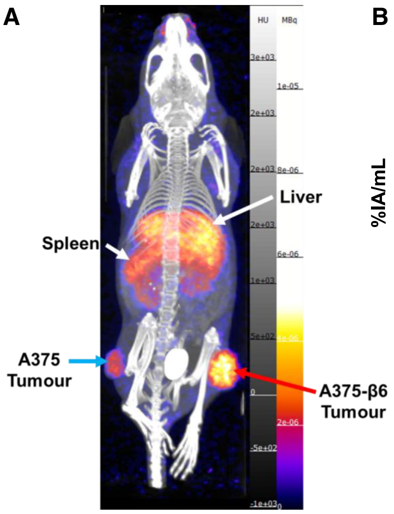

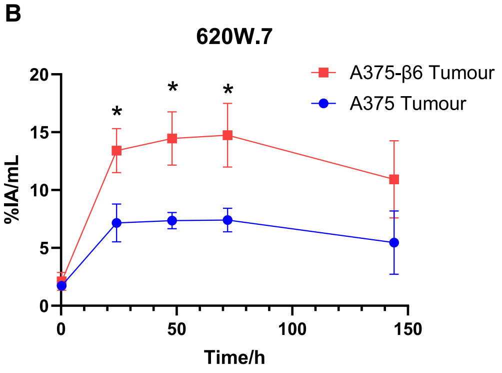

C

Time (h)	0.33	24	48	72	144
A375-β6/A375 relative uptake	1.22	1.87	1.96	1.99	2.00

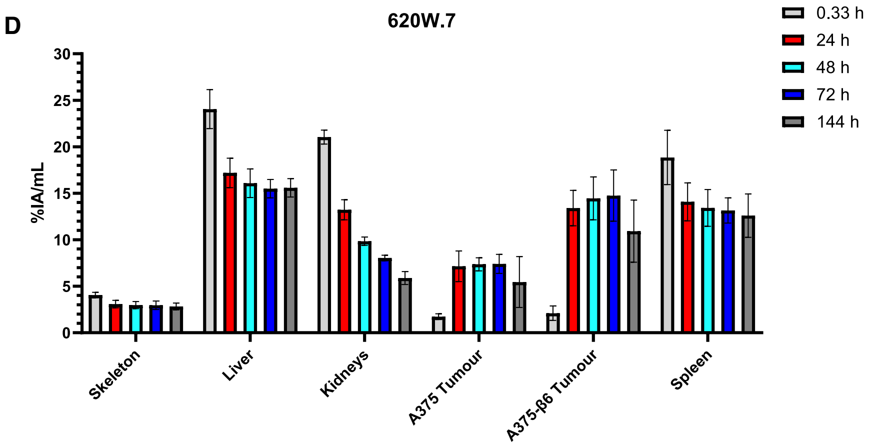

Figure 4. *In vivo* biodistribution of ^{89}Zr-620W.7
(A) Representative overlaid MIP views of PET/CT scans for a mouse injected with ^{89}Zr-620W.7, obtained after 72 h. (B) Comparison of radioactivity in A375-β6 and A375 tumors for ^{89}Zr-620W.7-injected mice (0.33h: n = 3 for A375-β6 and n = 4 for A375 tumor groups, 24–144 h: n = 4 for both groups). (C) Relative uptake of ^{89}Zr-620W.7 in A375-β6 tumors over A375 tumors. (D) Radiotracer uptake in organs of interest for ^{89}Zr-620W.7-injected mice (tumor n numbers as in (B), all other regions n = 5). Data are presented as mean ± standard deviation. *adjusted p value<0.033.

lymphatic drainage can result in the non-specific accumulation of high molecular weight biologics within tumors.[34]

^{89}Zr-620W.7 off-target uptake was seen in both the liver and spleen, a pattern characteristic of antibody-based imaging agents owing to their interaction with Fc-γ-receptors on immune cells within these sites. Glycoengineering of antibody Fc regions has previously been explored as an effective strategy through which to diminish uptake via this mechanism.[35] Hence, it may be possible to optimize the structure of 620W.7 to further improve its potential to act as a novel PET-imaging tracer, or alternatively as a targeted therapeutic agent as part of antibody-drug or radionuclide conjugates. Indeed, it is possible to envisage a scenario in which A20 peptides and 620W.7 could form a theranostic partnership, with the rapid trafficking of the peptide-based radiotracer facilitating diagnosis of αvβ6-expressing tumors that may be amenable to subsequent therapeutic intervention using 620W.7-targeted agents.

^{89}Zr-Ad5.KO1.A20 uptake was low in both A375 and A375-β6 tumors (Figure 6C). This was despite Ad5.KO1.A20 having demonstrated a capacity to selectively bind αvβ6-expressing cell lines *in vitro* (Figures 2, S2, and S3), suggesting that its failure to traffic to A375-β6 tumor xenografts relates to suboptimal *in vivo* stability. While A20 has previously been reported to suffer from protease-mediated degradation upon exposure to serum,[36–38] we did not observe a decrease in the ability of the Ad5.KO1.A20 protein to bind A375-β6 cells following its incubation in murine serum at 37°C (Figure S2). Levels of liver and renal sequestration were notably both quite high for this tracer (Figures 5E–5G), and its excretion rate was faster than for the 620W.7 and streptavidin-A20 tracers (Figure 6). It is possible that minor contributions by each of these factors combined to result in the overall lack of *in vivo* tumor accumulation seen for Ad5.KO1.A20. Alternatively, another presently unexplored clearance mechanism may underlie this observation. Greater insights into this may be gained by performing high-performance liquid chromatography on the blood and urine of ^{89}Zr-Ad5.KO1.A20-injected mice to evaluate its metabolism into breakdown products and clearance kinetics.

The present study was conducted using immunodeficient mice bearing human tumor xenografts, which prevented us from evaluating the impact of interactions with the immune system on the biodistribution of the investigated agents. Additional studies confirming retention of the αvβ6-selective trafficking properties of 620W.7 and streptavidin-A20 in the presence of intact immune system would further strengthen their translational potential. Furthermore, therapeutic use of the rat monoclonal 620W.7 antibody would require its humanization such as via engraftment of its complementarity-determining regions onto a human scaffold to reduce its immunogenicity. Nevertheless, the results of this study indicate that 620W.7 and streptavidin-A20 could form the basis of highly effective αvβ6-imaging agents. Future studies could also seek to examine whether therapeutic benefit may be gained by utilizing the agents to selectively deliver therapeutic payloads to αvβ6-expressing tumors as part of targeted antibody/peptide-drug conjugates.

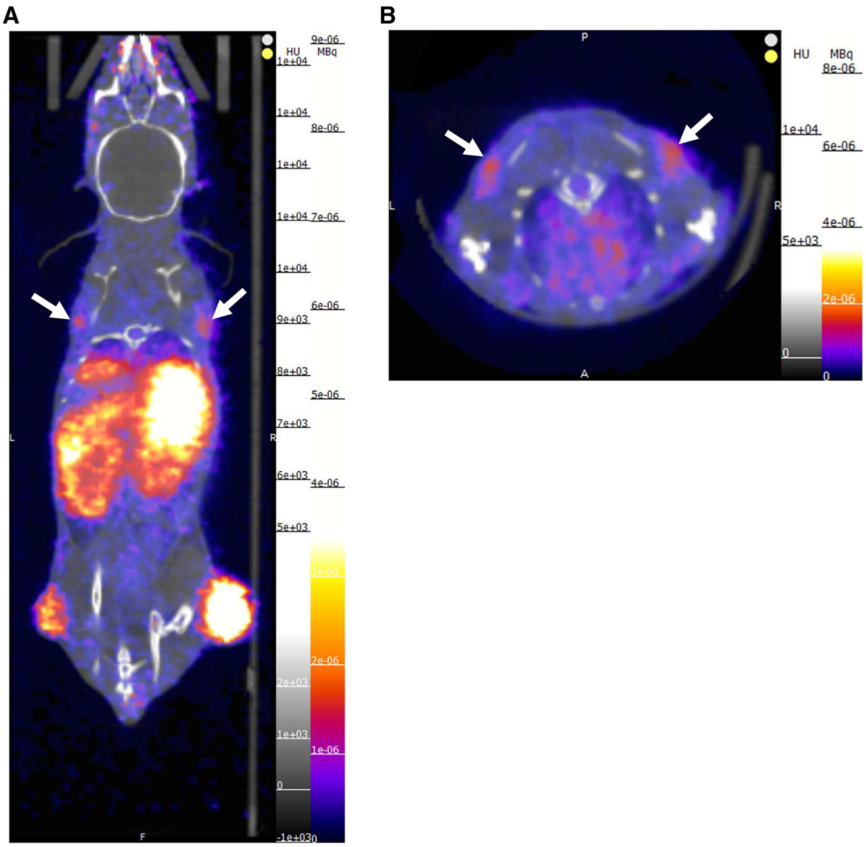

Figure 5. Axillary lymph node uptake of ^{89}Zr-620W.7

(A) Coronal and (B) transverse sections of an ^{89}Zr-620W.7-injected mouse, obtained after 48 h. White arrows denote radiotracer uptake within axillary lymph nodes.

(Radio) SDS-PAGE analysis

1-5 μg protein was loaded into wells of 4%–12% Bis-Tris gels (Invitrogen, Carlsbad, California, USA) and run at 150 V for 1 h 15 min. Samples prepared under denaturing conditions were boiled at 95°C in the presence of 0.05 M DTT prior to loading. Protein bands were visualized using SimplyBlue SafeStain (Invitrogen). For Radio-SDS-PAGE, ^{89}Zr-labeled sample-containing lanes were excised from gels and analyzed using a Scan-RAM radio-TLC scanner (LabLogic, Sheffield, UK).

Protein modeling

In silico structural predictions for recombinant adenoviral fiber knob proteins were generated using AlphaFold-Multimer run via the ColabFold notebook (v.1.5.5).[39–41]

Protein sequence alignments

Protein sequence alignments were produced using the ClustalW algorithm in BioEdit (v.7.2.5).[42]

METHODS

Reagents

The A20 peptide (NAVPNLRGDLQVLAQKVART) was synthesized by Peptide Synthetics Peptide Protein Research Ltd (Bishops Waltham, UK) with bi-terminal PEG-6 linkers and an N-terminal biotin modification to >95% purity. Streptavidin was purchased from Invitrogen (Rockford, Illinois, USA). The rat anti-human αvβ6 antibody clone 620W.7 was provided by John Marshall (Barts Cancer Institute, London, UK).

Production of recombinant fiber knob proteins

Sequences encoding adenoviral fiber knob proteins were synthesized and subcloned into pQE-30 expression vectors by GENEWIZ (Leipzig, Germany). Cultures of SG13009 *E.coli* harboring the relevant plasmid were grown to an OD_{600} of 0.6, and recombinant protein expression induced via addition of isopropyl-b-d-thiogalactopyranoside to a final concentration of 0.5 mM. Culture was continued for 16 h at 20°C and 6xHis-tagged protein purified from clarified bacterial lysates under native conditions using Ni-NTA agarose resin (QIAGEN, Hilden, Germany). Protein was gradient eluted as 1 mL fractions in buffers containing 50, 100, 200, and 300 mM imidazole in addition to 50 mM Tris, 300 mM NaCl, and 1 mM β-mercaptoethanol (pH 8.0).

Cell lines

All cell lines were maintained at 37°C in a humidified 5% CO_2 atmosphere. A375 and A375-β6 cells were provided by John Marshall (Bart's Cancer Institute, London, UK) and cultured in DMEM (Sigma-Aldrich, St. Louis, Missouri, USA), while BT-20 cells were purchased from American Type Culture Collection (ATCC, Manassas, Virginia, USA) and grown in MEM α (Sigma-Aldrich). Both cell lines were authenticated by short tandem repeat profiling conducted by Northgene (Deeside, UK). 4T1-β6 cells were generated in-house via MMLV-mediated overexpression of the murine β6 subunit in cells obtained from Awen Gallimore and Andrew Godkin (Cardiff University, UK) and grown in RPMI-1640 (Sigma-Aldrich). Culture media was supplemented with 10% FBS (20% for 4T1-β6 cells), 2% penicillin-streptomycin, and 1% L-glutamine (all Sigma-Aldrich).

Cell surface receptor staining

2×10^5 cells of the relevant cell line were incubated in 100 μL of 620W.7 (5 μg/mL), anti-αvβ6 antibody (clone 10D5, 2–5 μg/mL, [Merck Milipore, Burlington, Massachusetts, USA]), or relevant isotype controls (Rat IgG1 Kappa [RTK2071, BioLegend, Sn Diego, California, USA] or Mouse IgG2a kappa [MG2a-53, Abcam, Cambridge, UK]) on ice for 1 h, then washed twice prior to addition of 100 μL of relevant secondary antibody solution (Alexa Fluor 488

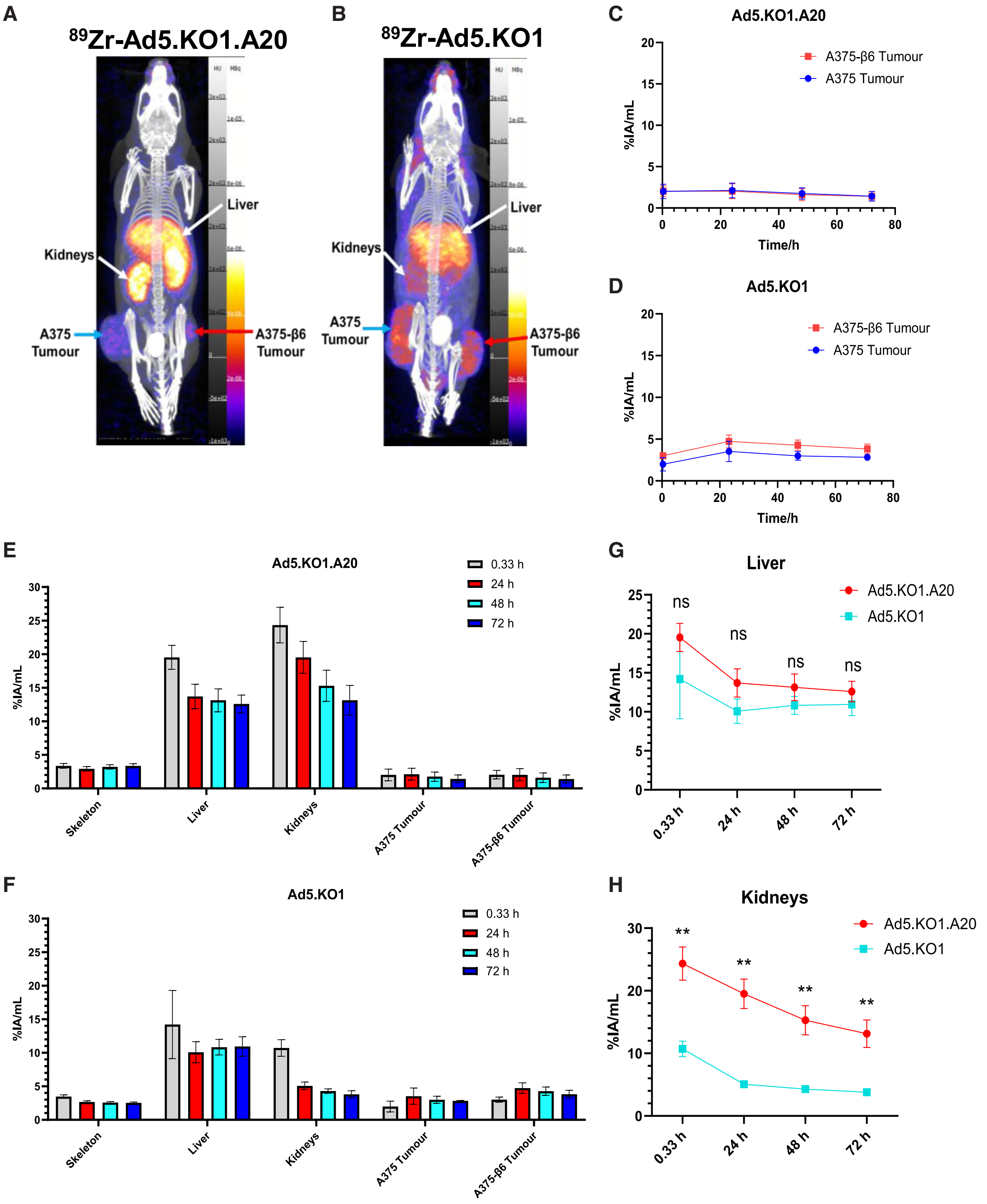

Figure 6. *In vivo* biodistribution of adenoviral fiber knob-based radiotracers

Representative overlaid MIP views of PET/CT scans for mice injected with (A) ^{89}Zr-Ad5.KO1.A20 and (B) ^{89}Zr-Ad5.KO1, obtained after 24 h. Comparison of radioactivity in A375-β6 and A375 tumors for (C) ^{89}Zr-Ad5.KO1.A20-injected mice (0.33 h: n = 3 for A375 and n = 2 for A375-β6 tumor groups, 24–72 h: n = 3 for both groups), and (D) ^{89}Zr-Ad5.KO1-injected mice (0.33–24 h: n = 3 for A375 and n = 4 for A375-β6 tumor groups, 48–72 h: n = 2 for A375 and n = 3 for A375-β6). Radiotracer uptake in organs of interest for (E) ^{89}Zr-Ad5.KO1.A20-injected mice (tumor n numbers as in C all other regions, n = 4) and (F) ^{89}Zr-Ad5.KO1-injected mice (tumor n numbers as in D all other regions, 0.33–24 h: n = 5, 48–72 h: n = 4). Comparison of levels of (G) hepatic and (H) renal tracer uptake for the ^{89}Zr-Ad5.KO1 (blue) and ^{89}Zr-Ad5.KO1.A20 (red) radiotracers (n numbers as in C and D). Data are mean ± standard deviation. ** adjusted p value < 0.0021, ns: no significant difference

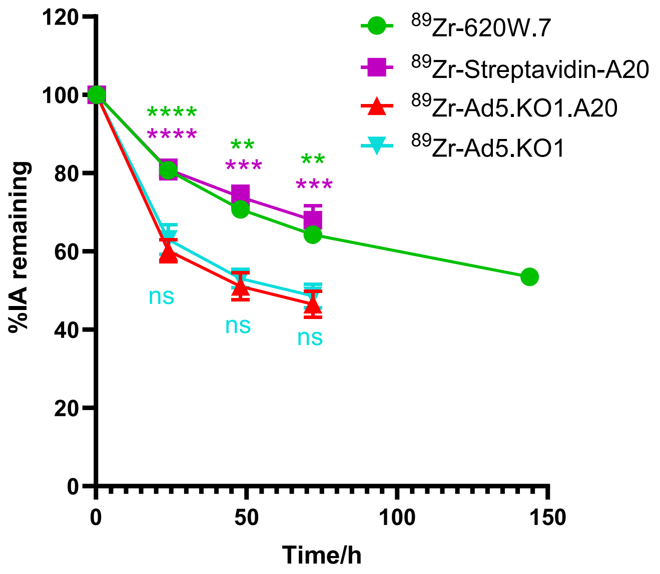

Figure 7. Excretion rates for αvβ6-targeted agents

The average radioactivity remaining at each time point was calculated by summing the total radioactivity present within whole body PET scans and expressed as a decay-corrected percentage of the activity present in initial scans for mice injected with ^{89}Zr-620W.7 (green, $n = 5$), ^{89}Zr-streptavidin-A20 (purple, 0.33–48 h: $n = 6$, 72 h: $n = 4$), ^{89}Zr-Ad5.KO1.A20 (red, $n = 4$) and ^{89}Zr-Ad5.KO1 (blue, 0.33–24 h: $n = 5$, 48–72 h: $n = 4$). Data are presented as mean ± standard deviation. **adjusted p value<0.0021, ***adjusted p value < 0.0002, ****adjusted p value < 0.0001, ns: no significant difference (compared to ^{89}Zr-Ad5.KO1.A20 group).

goat anti-rat [#Poly4054, BioLegend] or Alexa Fluor 647 goat anti-mouse [#A21237, Life Technologies, Eugene, Oregon, USA]). Cells were incubated with secondary antibodies in the dark for 30 min on ice, washed three times, and then fixed in 2% paraformaldehyde (Sigma-Aldrich). 10,000 single live cell events were acquired using a BD Accuri C6 plus instrument (BD Biosciences, Franklin Lakes, New Jersey, USA). Data analysis was performed using FlowJo (v.10.10.0, BD Biosciences).

In vitro binding assays

10-fold serial dilutions of αvβ6-targeted agents were prepared in PBS from 0.01 to 1,000 nM and incubated with 1 × 10^5 A375 or A375-β6 cells on ice for 30 min. Cells were washed three times, then binding assessed in flow cytometry-based assays as outlined previously. Fiber knob binding was detected using an anti-6xHisTag antibody (clone HIS.H8, 3.3 μg/mL, [antibodies.com, Cambridge, UK]), while an anti-streptavidin antibody (clone 3A20.2, 2 μg/mL, [BioLegend]) was used to assess binding of the streptavidin-A20 complex, both followed by secondary staining with Alexa Fluor 647 goat anti-mouse antibody (#A21237, Life Technologies). 620W.7 was detected using a goat anti-rat IgG Alexa Fluor 488 antibody (1 μg/mL, #Poly4054, BioLegend).

For assessment of agent stability in murine serum, samples were diluted in PBS or pooled serum from 9 to 10-week-old female CD-1 nude mice to a final concentration of 1 μM and incubated at 37°C for 0.5–48 h. Samples were diluted 10-fold in PBS prior to use in binding assays.

^{89}Zr-radiolabelling

^{89}Zr-radiolabelling was performed according to the methods of Vosjan et al.,[43] adapted for smaller scale reactions.[44] For streptavidin only, an additional succinylation step was performed following conjugation of the *p*-SCN-Bn-deferoxamine chelator (Macrocyclics, Plano, Texas, USA) as described previously.[33] Mock-labeled samples were prepared by incubating DFO-conjugated proteins with $^{nat}ZrCl_4$ (Sigma-Aldrich, St. Louis, Missouri, USA), prepared in 1 M oxalic acid to radiological concentrations, as opposed to [^{89}Zr] during the final incubation.

Radio-thin layer chromatography

4 μL of ^{89}Zr-labeled proteins were spotted onto baselines of solid phase glass microfiber chromatography paper strips (Agilent Technologies, Santa Clara, California, USA) and allowed to air dry prior to being placed into 50 mM DTPA eluent (pH 7.4). Strips were left in the mobile phase until the solvent front reached 105 mm, then analyzed using a Scan-RAM radio-TLC scanner with Laura radio-chromatography data analysis software (LabLogic).

Longitudinal micro-PET imaging and data analysis

5–6-week-old female CD-1 nude mice were purchased from Charles River Laboratories. All experiments were conducted in accordance with the UK Home Office regulations (ASPA 1986) under project license PBEB09FBB, with local approval from the Cardiff University Animal Welfare and Ethics Committee. 2 × 10^6 A375 or A375-β6 cells were subcutaneously injected bilaterally into the left and right hip regions of mice respectively and allowed to grow for 19 days. 2 MBq of the relevant radiotracer was then injected via the tail vein in a total volume of 20 μL ($n = 5$ or 6 mice per group). 100 μL/mouse iopamidol CT contrast agent was further administered intraperitoneally prior to imaging (Niopam 300, Bracco, Milan, Italy). 1 h Micro-PET scans were performed on 5% isoflurane in O_2 (1.5 L/min)-anaesthetized mice placed in a prone position in a nanoScan 122S PET/CT scanner (Mediso, Budapest, Hungary) at 0.33, 24, 48, and 72 h post-radiotracer injection. An additional scan was performed after 144 h for the 620W.7 group only. PET scans were followed immediately by 3-min CT scans, and PET/CT scans reconstructed with Nucline software (v,3.04, Mediso, Budapest, Hungary) using the Tera-Tomo-3D reconstruction algorithm with 4 iterations of 6 subsets and a 0.4 mm voxel size. Attenuation and scatter corrections were applied.

Data analysis performed using VivoQuant (v.4.0 patch 3, Invicro, Needham, Massachusetts, USA). Decay corrected total radioactivity within each region was expressed as a percentage of the initial injected activity for each mouse and normalized to region of interest volume (% injected activity/mL, %IA/mL). Only mice that underwent successful radiotracer tail vein injections were included in downstream analysis. Furthermore, tumors were excluded from biodistribution analysis if these could not be reliably selected as regions

of interest within overlaid PET/CT images. Some mice had to be culled prior to the end of the study owing to humane endpoints being reached. *n* numbers are indicated in figure legends.

Calculation of radiotracer excretion rates

Radiotracer excretion rates were determined by plotting the sum of decay-corrected radioactivity contained within whole body PET scans for each mouse, expressed as a percentage of the total activity present in the first scan.

Ex vivo dosimetry

Radioactivity measurements for organs and tumors harvested from mice culled following the final imaging time point were made using a sodium iodide well scintillation detector (Nuclear Enterprises, Reading, UK) and normalized to tissue mass to obtain % injected activity/g (%ID/g) values.

Statistical analysis

Statistical analysis was performed in GraphPad prism (v.10.4.1, Dotmatics, San Diego, California, USA). Non-linear regression curves were fitted using the specific binding with Hill slope equation. Comparison of radiotracer uptake within A375 and A375-β6 tumors was performed by fitting a mixed-model with Geisser-Greenhouse correction and Šídák's multiple comparisons post-hoc test. Statistical analysis of excretion rates was performed as described previously, with Dunnett's post-hoc test. Comparison of uptake of the radiotracers into tumor xenografts and organs of interest across the *in vivo* imaging time course was performed by conducting area-under-curve analysis followed by Welch's ANOVA with Dunnett's T3 multiple comparisons test. *Ex vivo* tumor and organ dosimetry measurements were compared using Welch's ANOVA with Dunnett's T3 multiple comparisons test. *adjusted p value < 0.0332, **adjusted p value < 0.0021, ***adjusted p value < 0.0002, ****adjusted p value<0.0001, ns: no significant difference.

DATA AND CODE AVAILABILITY

The datasets generated during and/or analyzed during the current study are available from the corresponding author on reasonable request.

ACKNOWLEDGMENTS

Schematics were produced in Biorender.com (Toronto, Ontario, Canada).

Funding was provided by a Cardiff University Innovation for All Project Grant awarded to R.J.B. and Wales Applied Virology Unit (WAVU). E.A.S. and R.J.B. were supported by a Cancer Research UK Biotherapeutic Program Grant awarded to A.L.P. (C52915/A29104).

The authors are grateful to Dr. Carly Bliss for providing access to CD-1 nude mouse serum, Dr. Joanna Zabkiewicz for useful discussions and Dr. Alicia Teijeira-Crespo for generation of 4T1-β6 cells.

AUTHOR CONTRIBUTIONS

All authors contributed to the study conception and design. Material preparation, data collection, and analysis were performed by E.A.S, S.J.P., and R.J.B. Animal experiments were performed by S.J.P. and R.J.B. The first draft of the manuscript was written by E.A.S. and all authors commented on previous versions of the manuscript. All authors read and approved the final manuscript.

DECLARATION OF INTERESTS

A.L.P. is CSO of Trocept Therapeutics, part of Accession Therapeutics Ltd. Accession Therapeutics Ltd had no involvement in the design or execution of this study.

SUPPLEMENTAL INFORMATION

Supplemental information can be found online at https://doi.org/10.1016/j.omton.2026.201236.

REFERENCES

1. Breuss, J.M., Gallo, J., DeLisser, H.M., Klimanskaya, I.V., Folkesson, H.G., Pittet, J.F., Nishimura, S.L., Aldape, K., Landers, D.V., Carpenter, W., et al. (1995). Expression of the β6 integrin subunit in development, neoplasia and tissue repair suggests a role in epithelial remodeling. J. Cell Sci. *108*, 2241–2251. https://doi.org/10.1242/jcs.108.6.2241.
2. Bandyopadhyay, A., and Raghavan, S. (2009). Defining the role of integrin alphavbeta6 in cancer. Curr. Drug Targets *10*, 645–652. https://doi.org/10.2174/138945009788680374.
3. Munger, J.S., Huang, X., Kawakatsu, H., Griffiths, M.J., Dalton, S.L., Wu, J., Pittet, J.F., Kaminski, N., Garat, C., Matthay, M.A., et al. (1999). The integrin alpha v beta 6 binds and activates latent TGF beta 1: a mechanism for regulating pulmonary inflammation and fibrosis. Cell *96*, 319–328. https://doi.org/10.1016/s0092-8674(00)80545-0.
4. Annes, J.P., Rifkin, D.B., and Munger, J.S. (2002). The integrin αVβ6 binds and activates latent TGFβ3. FEBS Lett. *511*, 65–68. https://doi.org/10.1016/S0014-5793(01)03280-X.
5. Li, H.X., Zheng, J.H., Fan, H.X., Li, H.P., Gao, Z.X., and Chen, D. (2013). Expression of αvβ6 integrin and collagen fibre in oral squamous cell carcinoma: association with clinical outcomes and prognostic implications. J. Oral Pathol. Med. *42*, 547–556. https://doi.org/10.1111/jop.12044.
6. Reader, C.S., Vallath, S., Steele, C.W., Haider, S., Brentnall, A., Desai, A., Moore, K.M., Jamieson, N.B., Chang, D., Bailey, P., et al. (2019). The integrin αvβ6 drives pancreatic cancer through diverse mechanisms and represents an effective target for therapy. J. Pathol. *249*, 332–342. https://doi.org/10.1002/path.5320.
7. Bates, R.C., Bellovin, D.I., Brown, C., Maynard, E., Wu, B., Kawakatsu, H., Sheppard, D., Oettgen, P., and Mercurio, A.M. (2005). Transcriptional activation of integrin beta6 during the epithelial-mesenchymal transition defines a novel prognostic indicator of aggressive colon carcinoma. J. Clin. Investig. *115*, 339–347. https://doi.org/10.1172/jci23183.
8. Zhang, Z.Y., Xu, K.S., Wang, J.S., Yang, G.Y., Wang, W., Wang, J.Y., Niu, W.B., Liu, E.Y., Mi, Y.T., and Niu, J. (2008). Integrin alphanvbeta6 acts as a prognostic indicator in gastric carcinoma. Clin. Oncol. *20*, 61–66. https://doi.org/10.1016/j.clon.2007.09.008.
9. Ahmed, N., Riley, C., Rice, G.E., Quinn, M.A., and Baker, M.S. (2002). Alpha(v)beta(6) integrin-A marker for the malignant potential of epithelial ovarian cancer. J. Histochem. Cytochem. *50*, 1371–1380. https://doi.org/10.1177/002215540205001010.
10. Li, Z., Biswas, S., Liang, B., Zou, X., Shan, L., Li, Y., Fang, R., and Niu, J. (2016). Integrin β6 serves as an immunohistochemical marker for lymph node metastasis and promotes cell invasiveness in cholangiocarcinoma. Sci. Rep. *6*, 30081. https://doi.org/10.1038/srep30081.
11. Whilding, L.M., Halim, L., Draper, B., Parente-Pereira, A.C., Zabinski, T., Davies, D.M., and Maher, J. (2019). CAR T-Cells Targeting the Integrin αvβ6 and Co-Expressing the Chemokine Receptor CXCR2 Demonstrate Enhanced Homing and Efficacy against Several Solid Malignancies. Cancers (Basel) *11*, 674. https://doi.org/10.3390/cancers11050674.
12. Phanthaphol, N., Somboonpatarakun, C., Suwanchiwasiri, K., Chieochansin, T., Sujjitjoon, J., Wongkham, S., Maher, J., Junking, M., and Yenchitsomanus, P.-t. (2021). Chimeric Antigen Receptor T Cells Targeting Integrin αvβ6 Expressed on Cholangiocarcinoma Cells. Front. Oncol. *11*, 657868. https://doi.org/10.3389/fonc.2021.657868.
13. Krige, D., Khanolkar, R.C., Davies, J.A., Baker, A.T., Webb, T.A., Shahreza, S., Uusi-Kerttula, H.K., Williams, L.M., Henderson, E., Moore, S.L., et al. (2024). TROCEPT: An oncolytic virus platform engineered for αvβ6 integrin targeted tumor-localised expression of an immune checkpoint inhibitor following intravenous delivery. J. Clin. Oncol. *42*, e14579. https://doi.org/10.1200/JCO.2024.42.16_suppl.e14579.

14. Uusi-Kerttula, H., Davies, J.A., Thompson, J.M., Wongthida, P., Evgin, L., Shim, K.G., Bradshaw, A., Baker, A.T., Rizkallah, P.J., Jones, R., et al. (2018). Ad5(NULL)-A20: A Tropism-Modified, αvβ6 Integrin-Selective Oncolytic Adenovirus for Epithelial Ovarian Cancer Therapies. Clin. Cancer Res. *24*, 4215–4224. https://doi.org/10.1158/1078-0432.Ccr-18-1089.

15. Liang, B., Shahbaz, M., Wang, Y., Gao, H., Fang, R., Niu, Z., Liu, S., Wang, B., Sun, Q., Niu, W., et al. (2015). Integrinβ6-targeted immunoliposomes mediate tumor-specific drug delivery and enhance therapeutic efficacy in colon carcinoma. Clin. Cancer Res. *21*, 1183–1195. https://doi.org/10.1158/1078-0432.Ccr-14-1194.

16. Saha, A., Ellison, D., Thomas, G.J., Vallath, S., Mather, S.J., Hart, I.R., and Marshall, J.F. (2010). High-resolution in vivo imaging of breast cancer by targeting the pro-invasive integrin αvβ6. J. Pathol. *222*, 52–63. https://doi.org/10.1002/path.2745.

17. Hausner, S.H., DiCara, D., Marik, J., Marshall, J.F., and Sutcliffe, J.L. (2007). Use of a peptide derived from foot-and-mouth disease virus for the noninvasive imaging of human cancer: generation and evaluation of 4-[18F]fluorobenzoyl A20FMDV2 for in vivo imaging of integrin alphavbeta6 expression with positron emission tomography. Cancer Res. *67*, 7833–7840. https://doi.org/10.1158/0008-5472.Can-07-1026.

18. Altmann, A., Sauter, M., Roesch, S., Mier, W., Warta, R., Debus, J., Dyckhoff, G., Herold-Mende, C., and Haberkorn, U. (2017). Identification of a Novel ITGαvβ6-Binding Peptide Using Protein Separation and Phage Display. Clin. Cancer Res. *23*, 4170–4180. https://doi.org/10.1158/1078-0432.Ccr-16-3217.

19. Flechsig, P., Lindner, T., Loktev, A., Roesch, S., Mier, W., Sauter, M., Meister, M., Herold-Mende, C., Haberkorn, U., and Altmann, A. (2019). PET/CT Imaging of NSCLC with a α(v)β(6) Integrin-Targeting Peptide. Mol. Imaging Biol. *21*, 973–983. https://doi.org/10.1007/s11307-018-1296-6.

20. Kimura, R.H., Wang, L., Shen, B., Huo, L., Tummers, W., Filipp, F.V., Guo, H.H., Haywood, T., Abou-Elkacem, L., Baratto, L., et al. (2019). Evaluation of integrin αvβ6 cystine knot PET tracers to detect cancer and idiopathic pulmonary fibrosis. Nat. Commun. *10*, 4673. https://doi.org/10.1038/s41467-019-11863-w.

21. Quigley, N.G., Steiger, K., Hoberück, S., Czech, N., Zierke, M.A., Kossatz, S., Pretze, M., Richter, F., Weichert, W., Pox, C., et al. (2022). PET/CT imaging of head-and-neck and pancreatic cancer in humans by targeting the "Cancer Integrin" αvβ6 with Ga-68-Trivehexin. Eur. J. Nucl. Med. Mol. Imaging *49*, 1136–1147. https://doi.org/10.1007/s00259-021-05559-x.

22. Hausner, S.H., Bold, R.J., Cheuy, L.Y., Chew, H.K., Daly, M.E., Davis, R.A., Foster, C.C., Kim, E.J., and Sutcliffe, J.L. (2019). Preclinical Development and First-in-Human Imaging of the Integrin α(v)β(6) with [(18)F]α(v)β(6)-Binding Peptide in Metastatic Carcinoma. Clin. Cancer Res. *25*, 1206–1215. https://doi.org/10.1158/1078-0432.Ccr-18-2665.

23. Keat, N., Kenny, J., Chen, K., Onega, M., Garman, N., Slack, R.J., Parker, C.A., Lumbers, R.T., Hallett, W., Saleem, A., et al. (2018). A Microdose PET Study of the Safety, Immunogenicity, Biodistribution, and Radiation Dosimetry of (18)F-FB-A20FMDV2 for Imaging the Integrin α(v)β(6). J. Nucl. Med. Technol. *46*, 136–143. https://doi.org/10.2967/jnmt.117.203547.

24. Roesch, S., Lindner, T., Sauter, M., Loktev, A., Flechsig, P., Müller, M., Mier, W., Warta, R., Dyckhoff, G., Herold-Mende, C., et al. (2018). Comparison of the RGD Motif–Containing αvβ6 Integrin–Binding Peptides SFLAP3 and SFITGv6 for Diagnostic Application in HNSCC. J. Nucl. Med. *59*, 1679–1685. https://doi.org/10.2967/jnumed.118.210013.

25. Chigoho, D.M., Bridoux, J., and Hernot, S. (2021). Reducing the renal retention of low- to moderate-molecular-weight radiopharmaceuticals. Curr. Opin. Chem. Biol. *63*, 219–228. https://doi.org/10.1016/j.cbpa.2021.06.008.

26. Kimura, R.H., Iagaru, A., and Guo, H.H. (2023). Mini review of first-in-human integrin αvβ6 PET tracers. Front. Nucl. Med. *3*, 1271208. https://doi.org/10.3389/fnume.2023.1271208.

27. Hausner, S.H., Bauer, N., Hu, L.Y., Knight, L.M., and Sutcliffe, J.L. (2015). The Effect of Bi-Terminal PEGylation of an Integrin $\alpha v\beta_6$-Targeted ^{18}F Peptide on Pharmacokinetics and Tumor Uptake. J. Nucl. Med. *56*, 784–790. https://doi.org/10.2967/jnumed.114.150680.

28. Jakubczak, J.L., Rollence, M.L., Stewart, D.A., Jafari, J.D., Von Seggern, D.J., Nemerow, G.R., Stevenson, S.C., and Hallenbeck, P.L. (2001). Adenovirus Type 5 Viral Particles Pseudotyped with Mutagenized Fiber Proteins Show Diminished Infectivity of Coxsackie B-Adenovirus Receptor-Bearing Cells. J. Virol. *75*, 2972–2981. https://doi.org/10.1128/jvi.75.6.2972-2981.2001.

29. Davies, J.A., Marlow, G., Uusi-Kerttula, H.K., Seaton, G., Piggott, L., Badder, L.M., Clarkson, R.W.E., Chester, J.D., and Parker, A.L. (2021). Efficient Intravenous Tumor Targeting Using the alphavbeta6 Integrin-Selective Precision Virotherapy Ad5NULL-A20. Viruses *13*, 864. https://doi.org/10.3390/v13050864.

30. Kogelberg, H., Tolner, B., Thomas, G.J., Di Cara, D., Minogue, S., Ramesh, B., Sodha, S., Marsh, D., Lowdell, M.W., Meyer, T., et al. (2008). Engineering a single-chain Fv antibody to alpha v beta 6 integrin using the specificity-determining loop of a foot-and-mouth disease virus. J. Mol. Biol. *382*, 385–401. https://doi.org/10.1016/j.jmb.2008.07.013.

31. Whilding, L.M., Parente-Pereira, A.C., Zabinski, T., Davies, D.M., Petrovic, R.M.G., Kao, Y.V., Saxena, S.A., Romain, A., Costa-Guerra, J.A., Violette, S., et al. (2017). Targeting of Aberrant αvβ6 Integrin Expression in Solid Tumors Using Chimeric Antigen Receptor-Engineered T Cells. Mol. Ther. *25*, 259–273. https://doi.org/10.1016/j.ymthe.2016.10.012.

32. Singh, A.N., McGuire, M.J., Li, S., Hao, G., Kumar, A., Sun, X., and Brown, K.C. (2014). Dimerization of a phage-display selected peptide for imaging of αvβ6- integrin: two approaches to the multivalent effect. Theranostics *4*, 745–760. https://doi.org/10.7150/thno.7811.

33. Wilbur, D.S., Hamlin, D.K., Buhler, K.R., Pathare, P.M., Vessella, R.L., Stayton, P.S., and To, R. (1998). Streptavidin in antibody pretargeting. 2. Evaluation Of methods for decreasing localization of streptavidin to kidney while retaining its tumor binding capacity. Bioconjug. Chem. *9*, 322–330. https://doi.org/10.1021/bc970182v.

34. Wu, J. (2021). The Enhanced Permeability and Retention (EPR) Effect: The Significance of the Concept and Methods to Enhance Its Application. J. Pers. Med. *11*, 771. https://doi.org/10.3390/jpm11080771.

35. Vivier, D., Sharma, S.K., Adumeau, P., Rodriguez, C., Fung, K., and Zeglis, B.M. (2019). The Impact of FcγRI Binding on Immuno-PET. J. Nucl. Med. *60*, 1174–1182. https://doi.org/10.2967/jnumed.118.223636.

36. Cardle, I.I., Jensen, M.C., Pun, S.H., and Sellers, D.L. (2021). Optimized serum stability and specificity of an αvβ6 integrin-binding peptide for tumor targeting. J. Biol. Chem. *296*, 100657. https://doi.org/10.1016/j.jbc.2021.100657.

37. Hung, K.-y., Kowalczyk, R., Desai, A., Brimble, M.A., Marshall, J.F., and Harris, P.W.R. (2022). Synthesis and Systematic Study on the Effect of Different PEG Units on Stability of PEGylated, Integrin-αvβ6-Specific A20FMDV2 Analogues in Rat Serum and Human Plasma. Molecules *27*, 4331. https://doi.org/10.3390/molecules27144331.

38. Hung, K.-y., Harris, P.W.R., Desai, A., Marshall, J.F., and Brimble, M.A. (2017). Structure-activity relationship study of the tumour-targeting peptide A20FMDV2 via modification of Lys16, Leu13, and N- and/or C-terminal functionality. Eur. J. Med. Chem. *136*, 154–164. https://doi.org/10.1016/j.ejmech.2017.05.008.

39. Jumper, J., Evans, R., Pritzel, A., Green, T., Figurnov, M., Ronneberger, O., Tunyasuvunakool, K., Bates, R., Žídek, A., Potapenko, A., et al. (2021). Highly accurate protein structure prediction with AlphaFold. Nature *596*, 583–589. https://doi.org/10.1038/s41586-021-03819-2.

40. Mirdita, M., Schütze, K., Moriwaki, Y., Heo, L., Ovchinnikov, S., and Steinegger, M. (2022). ColabFold: making protein folding accessible to all. Nat. Methods *19*, 679–682. https://doi.org/10.1038/s41592-022-01488-1.

41. Evans, R., O'Neill, M., Pritzel, A., Antropova, N., Senior, A., Green, T., Žídek, A., Bates, R., Blackwell, S., Yim, J., et al. (2021). Protein complex prediction with AlphaFold-Multimer. Preprint at bioRxiv, 10.04.463034. https://doi.org/10.1101/2021.10.04.463034.

42. Hall, T.A. (1999). BioEdit: a user-friendly biological sequence alignment editor and analysis program for windows. Nucleic Acids Symp. Ser *41*, 95–98.

43. Vosjan, M.J.W.D., Perk, L.R., Visser, G.W.M., Budde, M., Jurek, P., Kiefer, G.E., and van Dongen, G.A.M.S. (2010). Conjugation and radiolabeling of monoclonal antibodies with zirconium-89 for PET imaging using the bifunctional chelate p-isothiocyanatobenzyl-desferrioxamine. Nat. Protoc. *5*, 739–743. https://doi.org/10.1038/nprot.2010.13.

44. Knight, J.C., Paisey, S.J., Dabkowski, A.M., Marculescu, C., Williams, A.S., Marshall, C., and Cornelissen, B. (2016). Scaling-down antibody radiolabeling reactions with zirconium-89. Dalton Trans. *45*, 6343–6347. https://doi.org/10.1039/C5DT04774A.

OMTON, Volume *34*

Supplemental information

Comparison of the *in vivo* biodistributions of αvβ6-binding agents for PET imaging applications

Emma A. Swift, Stephen J. Paisey, Toby J. Phesse, John F. Marshall, Alan L. Parker, and Rebecca J. Bayliss

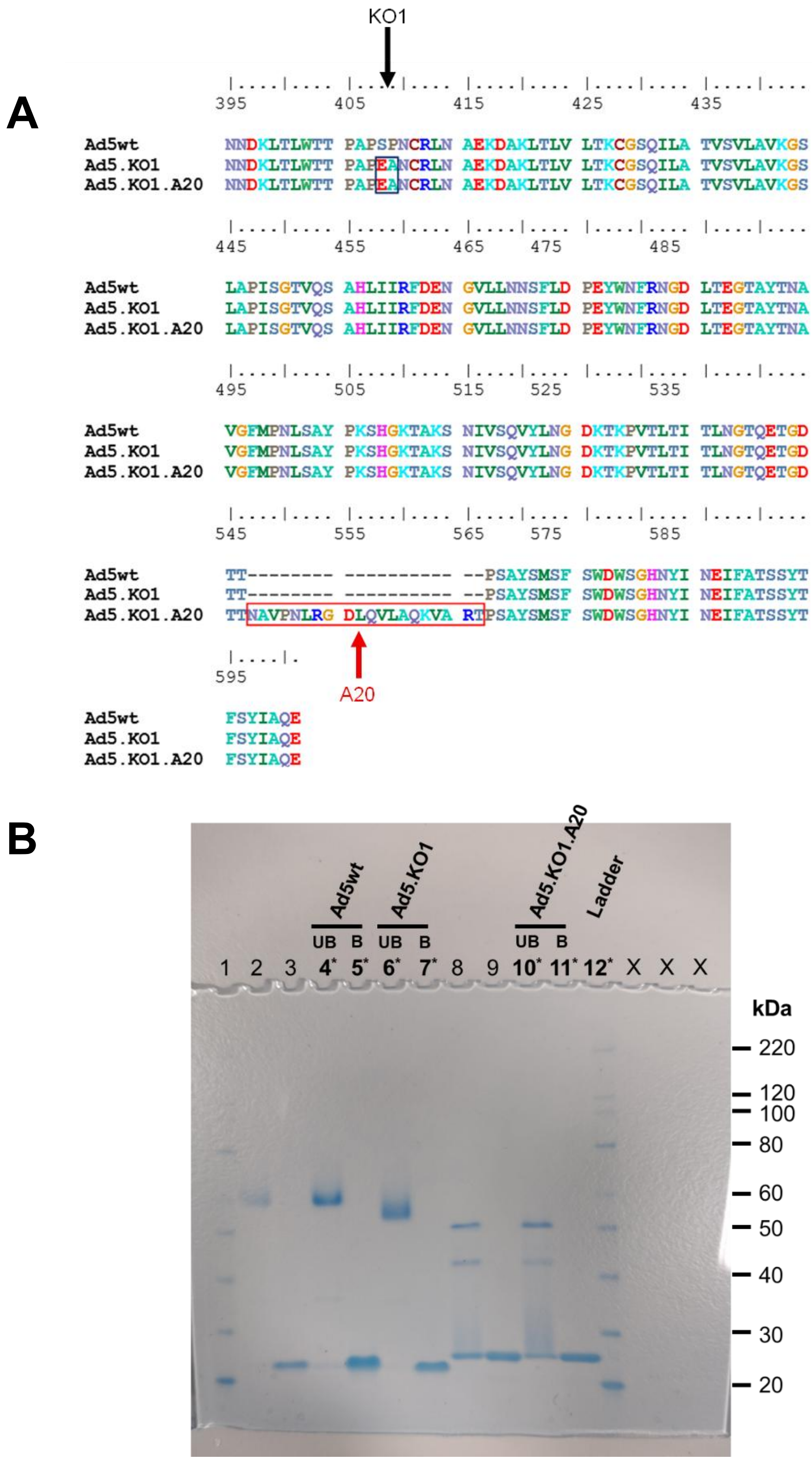

Figure S1. Fiber knob sequence alignments for the Ad5 wildtype (wt), Ad5.KO1 and Ad5.KO1.A20 fiber knob proteins. (A) The positions of the KO1 mutation (black box) and A20 peptide insert (red box) are indicated. Alignments were produced using the ClustalW algorithm in BioEdit v7.2.5. (B) Full gel image related to Figure 1C. Lanes included in Figure 1C are denoted in bold typeface with superscript asterisks. Lanes marked with an X were not loaded with sample.

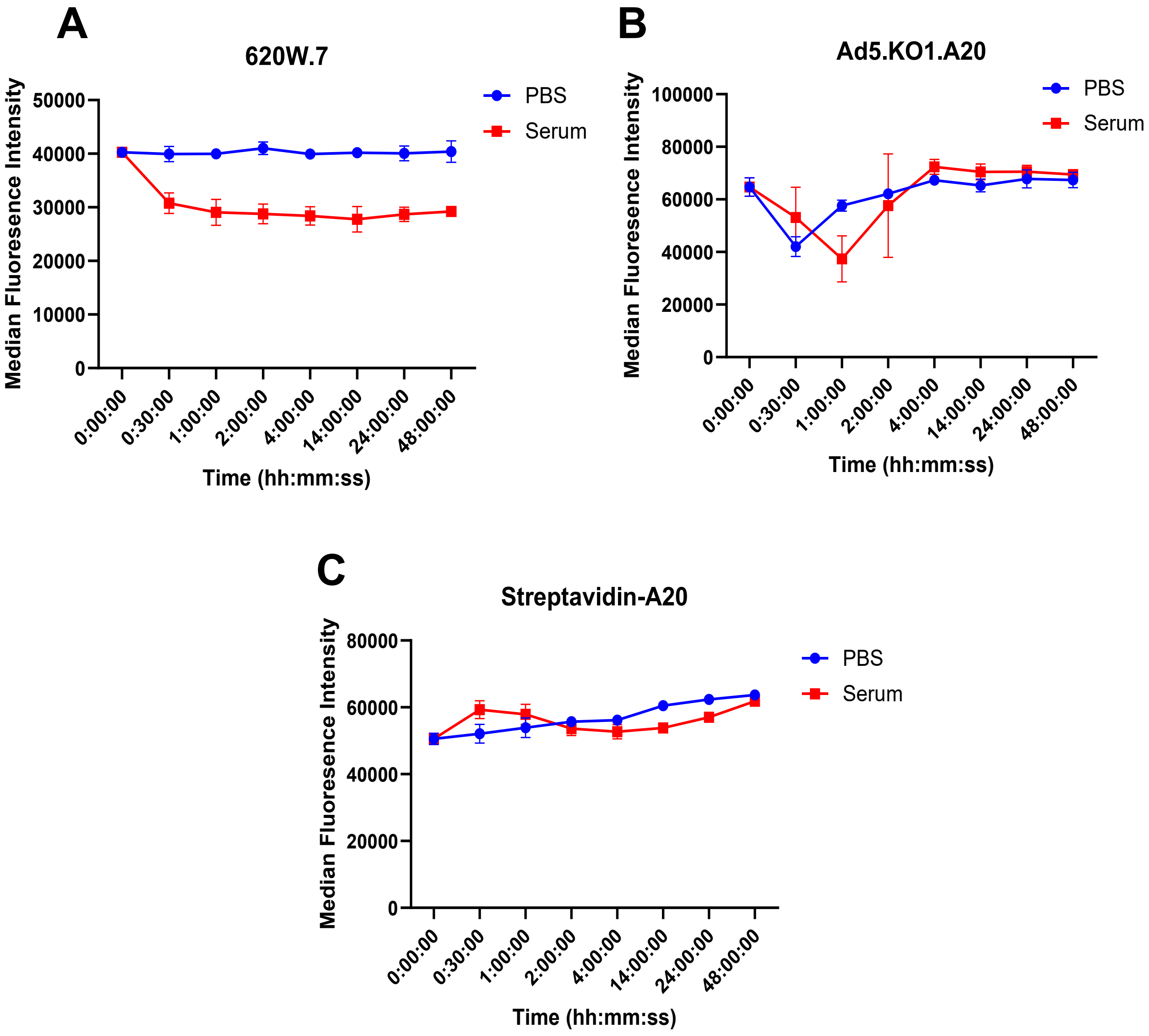

Figure S2. Serum stability of αvβ6-targeted agents. Binding of (A) 620W.7, (B) recombinant Ad5.KO1.A20 and (C) streptavidin-A20 samples to A375-β6 cells following their incubation in PBS (blue circles) or CD-1 nude mouse serum (red squares). Data are mean of three technical replicates ± standard deviation.

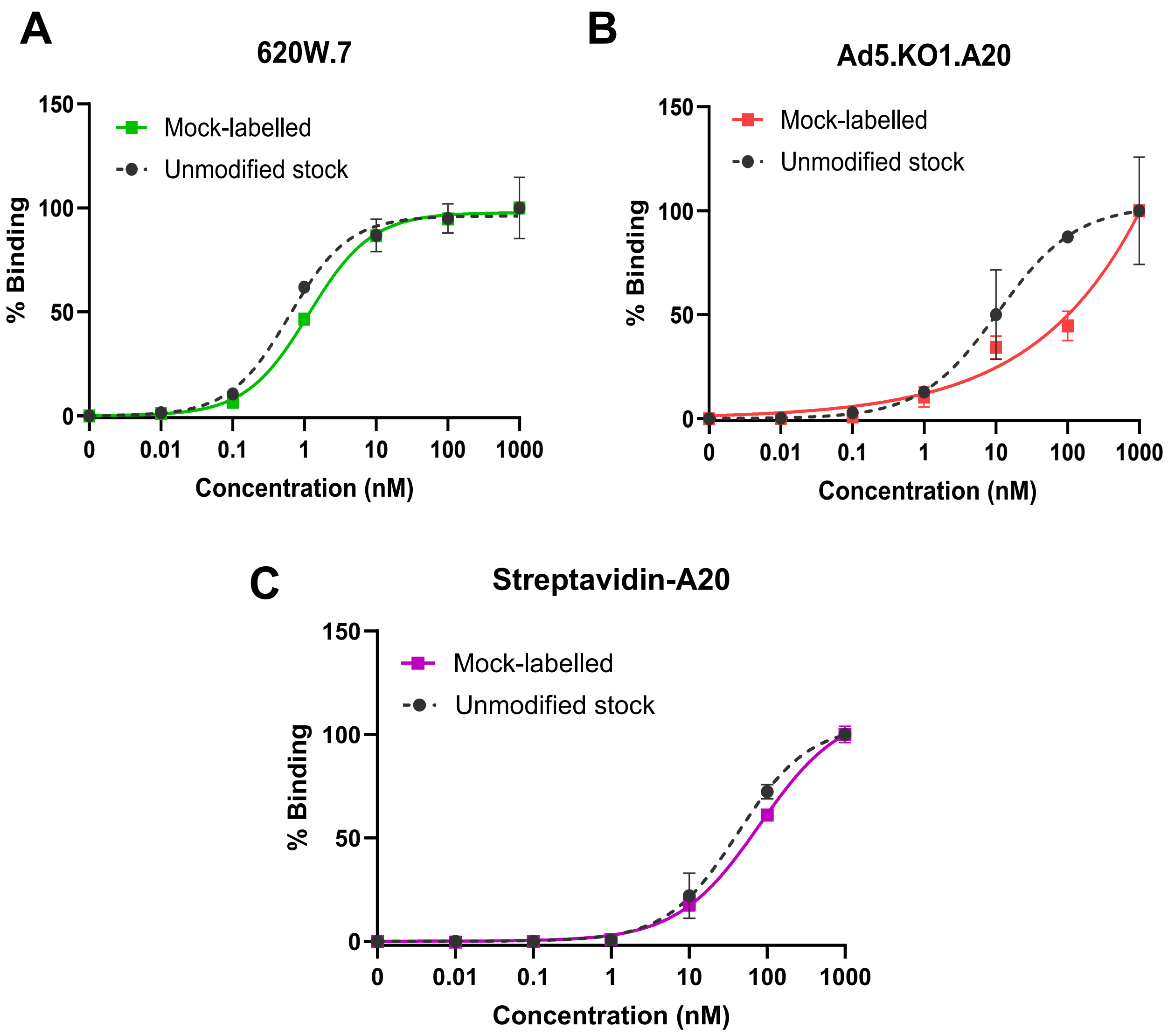

Figure S3. Comparison of unmodified and mock Zr-labelled αvβ6-targeted agent binding to A375-β6 cells. A375-β6 cells were incubated with increasing concentrations of (A) 620W.7, (B) Ad5.KO1.A20 fiber knob proteins, or (C) streptavidin-A20. Mock-labelled samples were prepared by incubating *p*-SCN-Bn-DFO conjugated samples with non-radioactive natural Zr isotopes. Mock-labelled streptavidin was subsequently incubated with biotinylated A20 in a 1:4 streptavidin:A20 molar ratio. Binding of mock-labelled samples (solid, coloured lines) was compared to unmodified stocks (dashed black lines) of each agent. 0% binding was defined as the MFI for the 0 nM condition, and 100% binding as the MFI for the 1000 nM concentration for each dataset. Data are presented as mean of 3 technical repeats ± standard deviation.

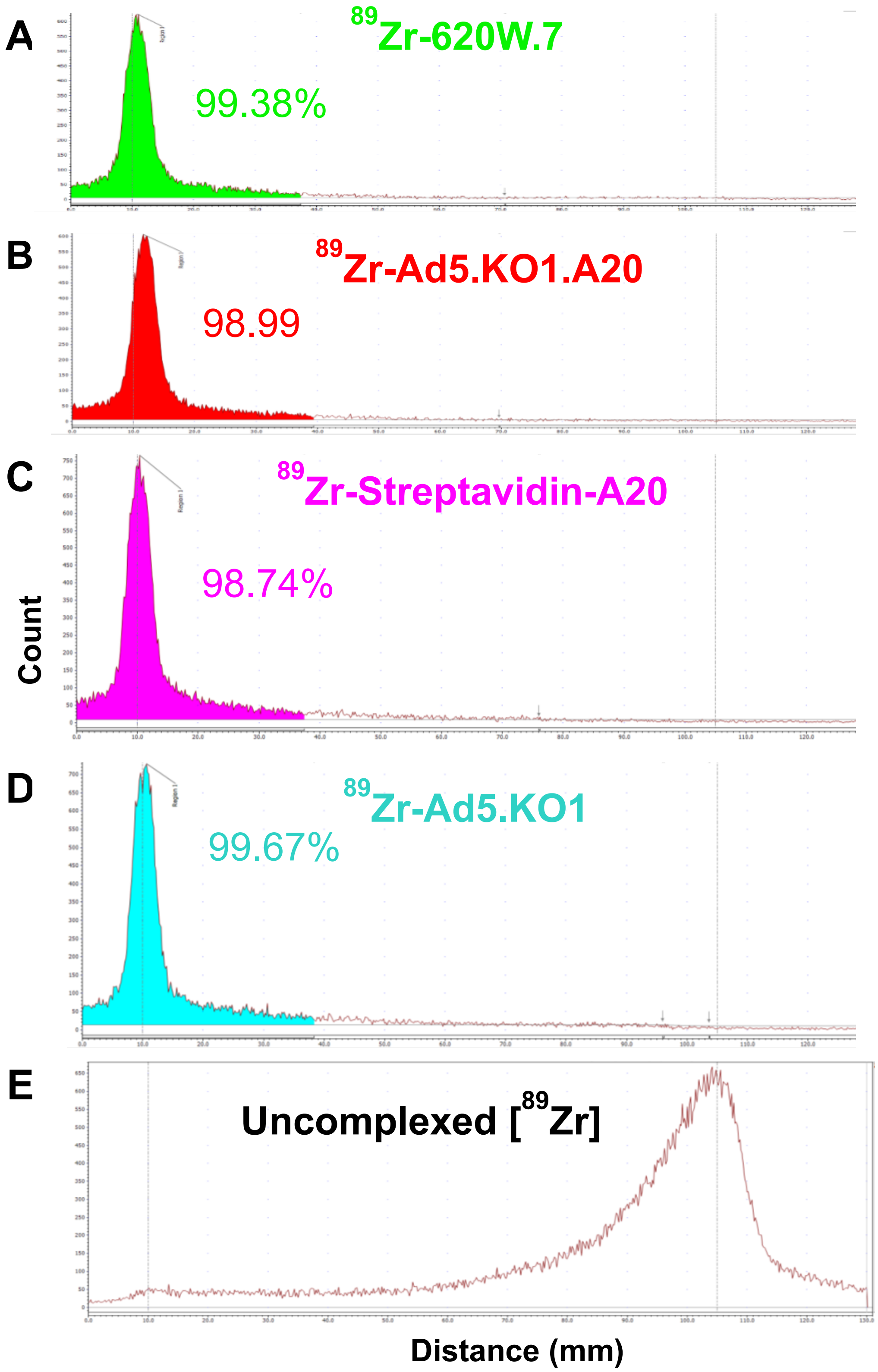

Figure S4. Evaluation of radiotracer yields by radio thin-layer chromatography. Radio-TLC chromatograms for (A) ^{89}Zr-620W.7, (B) ^{89}Zr-Ad5.KO1.A20, (C) ^{89}Zr-streptavidin-A20, and (D) ^{89}Zr-Ad5.KO1. Grey arrows indicate points on the trace selected to set the baseline. The area (% of total) contained within each of the highlighted peaks is indicated. (E) The radio-TLC trace for uncomplexed ^{89}Zr is shown for comparison.

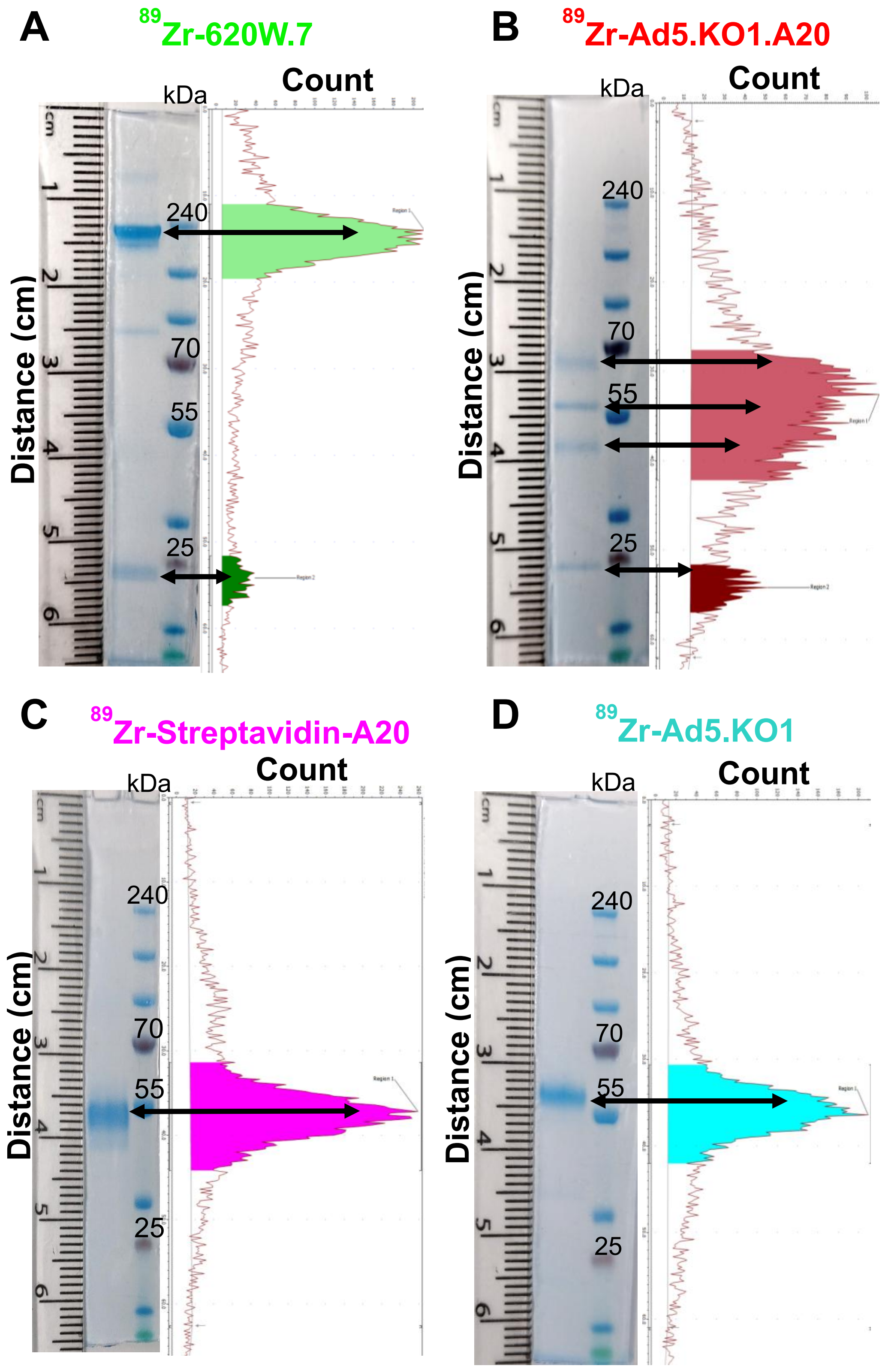

Figure S5. Assessment of radiotracer purity by radio-SDS-PAGE. Images of SDS-PAGE gels for (A) ^{89}Zr-620W.7, (B) ^{89}Zr-Ad5.KO1.A20, (C) ^{89}Zr-streptavidin-A20, and (D) ^{89}Zr-Ad5.KO1, with protein bands visualized by Coomassie blue staining, and corresponding radiochromatograms produced by analysing the relevant gel lane with a radio-TLC scanner.

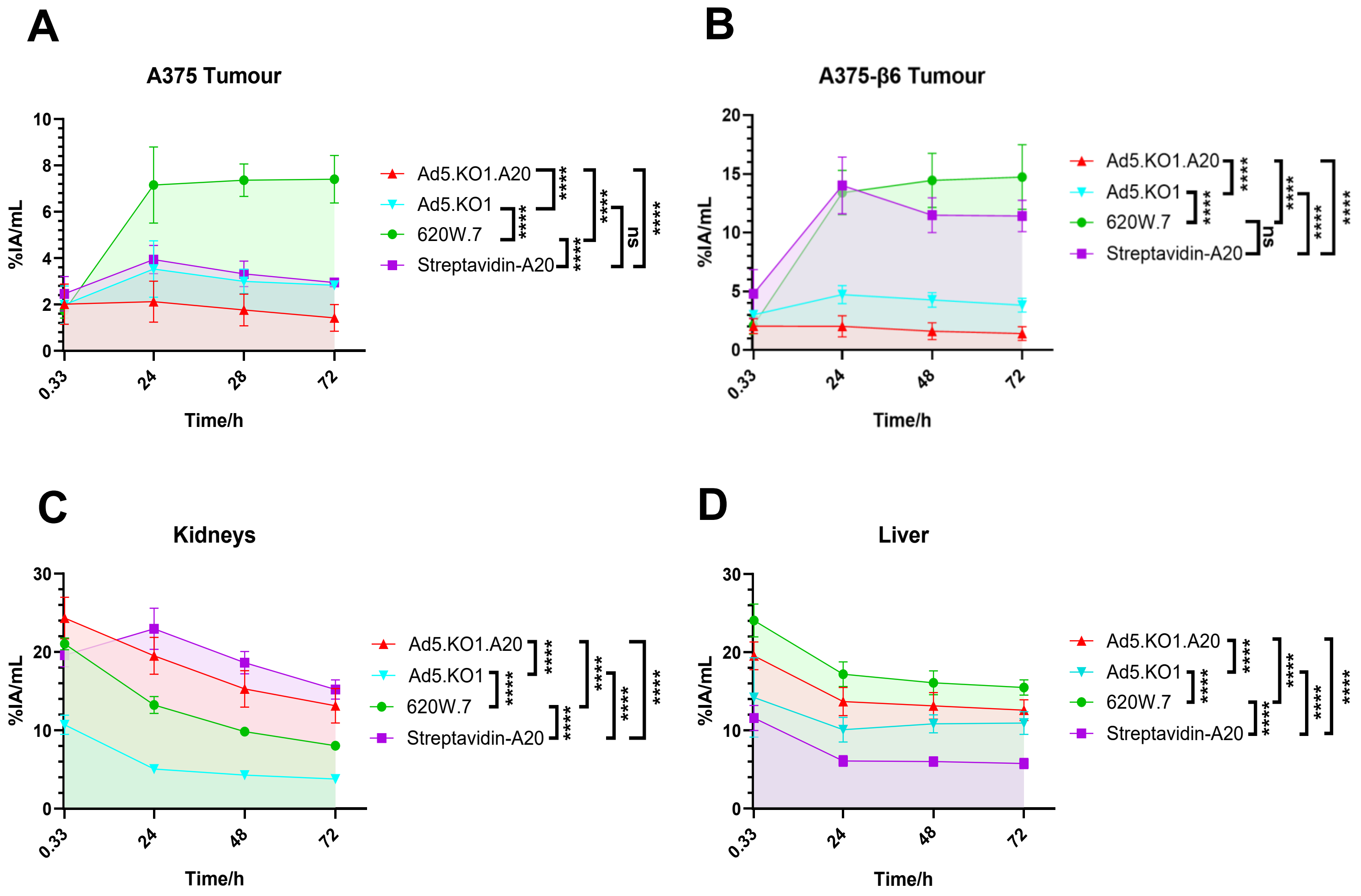

Figure S6. Comparison of the *in vivo* biodistributions of ^{89}Zr-620W.7, ^{89}Zr-Streptavidin-A20, ^{89}Zr-Ad5.KO1.A20 and ^{89}Zr-Ad5.KO1 radiotracers. Graphs depicting uptake of αvβ6-targeted agents into (A) A375 tumours (B) A375-β6 tumours (C) kidneys and (D) livers of mice injected with ^{89}Zr-labelled Ad5.KO1.A20 (red triangles), Ad5.KO1 (blue inverted triangles), 620W.7 (green circles) or streptavidin-A20 (purple squares) across the 0.33-72 h imaging timepoints. N numbers as in main text figures 5E (Ad5.KO1.A20), 5F (Ad5.KO1), 4D (620W.7), and 3D (streptavidin-A20). Data are presented as mean ± standard deviation. Statistical analysis was performed by comparing area-under-curve for each agent using Welch's ANOVA with Dunnett's T3 multiple comparisons test. ****adjusted p<0.0001, ns: no significant difference.

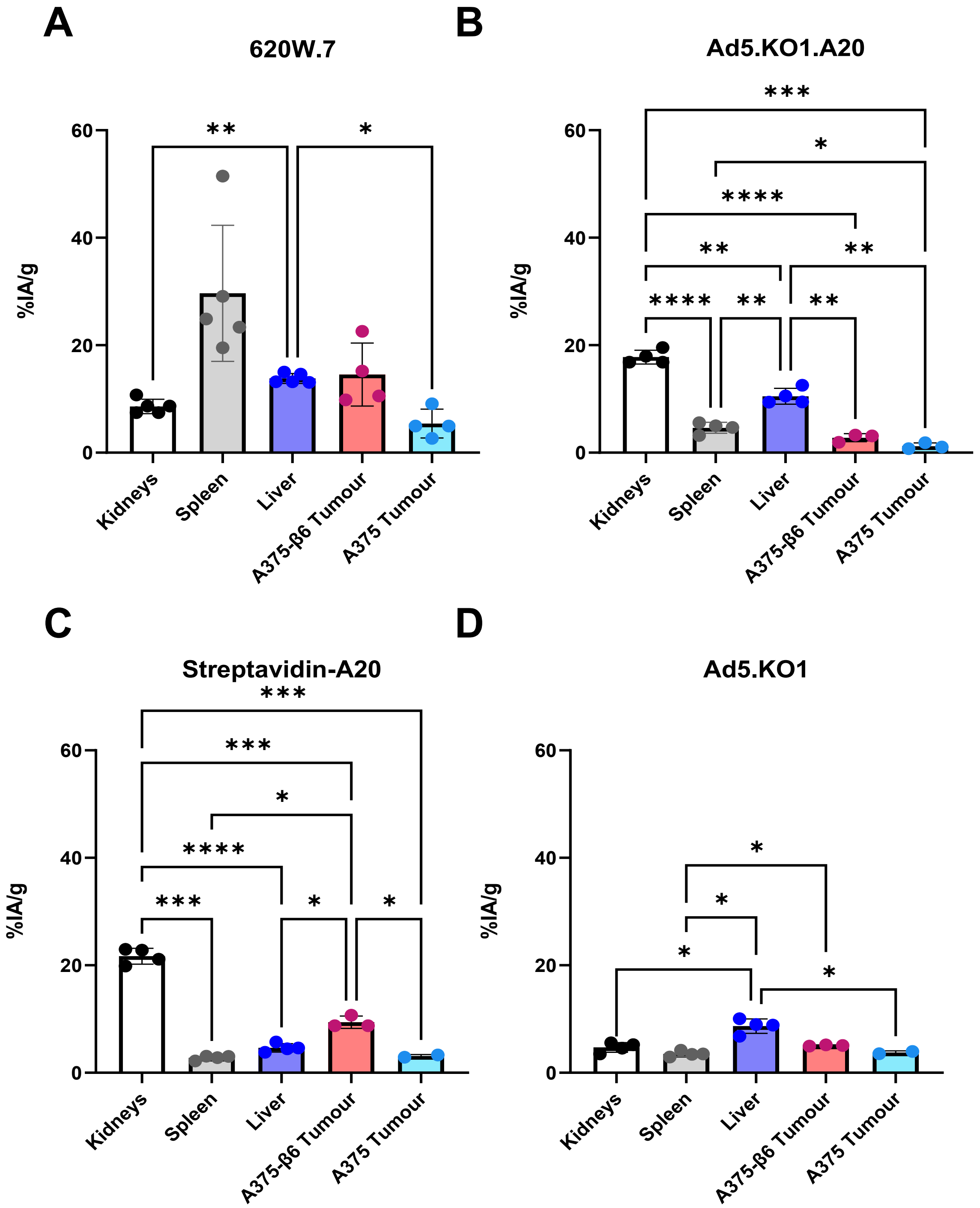

Figure S7. ***Ex vivo*** **biodistribution analysis.** Radioactivity contained within organs and tumours harvested from mice injected with (A) ^{89}Zr-620W.7 (n=5 for organs, n=4 for tumours), (B) ^{89}Zr-Ad5.KO1.A20 (n=4 for organs, n=3 for tumours), (C) ^{89}Zr-streptavidin-A20 (n=4 for organs, n=3 for A375-β6 tumours, n=2 for A375 tumours) and (D) ^{89}Zr-Ad5.KO1 (n=4 for organs, n=3 for A375-β6 tumours, n=2 for A375 tumours) following the final imaging timepoint for each agent (144 h post-injection for 620W.7, 72 h post-injection for the remaining agents). Data are presented as mean ± standard deviation. Statistical analysis was performed using Welch's ANOVA with Dunnett's T3 multiple comparisons test. *adjusted p<0.0332, **adjusted p<0.0021, ***adjusted p<0.0002, ****adjusted p<0.0001.

Video S1. Bladder and bowel-mediated radiotracer excretion videos. Time series videos generated by dividing micro-PET scans obtained at 0.33 h into 6x10 minute segments. High radioactivity levels are visible in the intestines and bladders of (A) ^{89}Zr-Ad5.KO1.A20 and (B) ^{89}Zr-Ad5.KO1-injected mice, but not equivalent regions of mice injected with (C) ^{89}Zr-streptavidin-A20 or (D) ^{89}Zr-620W.
